# Decision-theoretic recruitment and validation planning for depression surveys: An illustrative Chinese application

**DOI:** 10.1371/journal.pone.0359867

**Published:** 2026-10-01

**Authors:** Han Liu

**Affiliations:** Department of Behavioural Science and Health, Institute of Epidemiology and Public Health, University College London, London, United Kingdom; Nanjing University, CHINA

## Abstract

**Background:**

Survey planning must balance recruitment losses against uncertainty in screening yield, diagnostic accuracy and survey design. This study examined how these considerations determine invitation requirements and diagnostic-validation allocation.

**Methods:**

A Beta planning distribution used 170 PHQ-9-positive results among 1,045 Chinese community participants and an effective sample size of 100. Asymmetric recruitment loss selected conditional and joint-distribution invitation targets. A separate cost-plus-variance Bayes criterion jointly allocated survey clusters and independent reference-positive and reference-negative validation samples. Parameter uncertainty and future-data performance were evaluated by integration and simulation.

**Results:**

With an illustrative underplanning-to-overplanning loss ratio of 3, conditional planning selected a screening-positive proportion of 0.1862 and 4,794 screening invitations. Integrating design uncertainty increased this target to 5,346. Current-prevalence planning required 9,247 conditional invitations or 11,866 under joint parameter uncertainty when diagnostic accuracy was known within each scenario. The original validation sample imposed a reference-point half-width floor of 0.0292, exceeding the 0.020 target. Under specified relative costs, the joint allocation used 21,708 invitations and 240/4,538 reference-positive/reference-negative validation participants. Its prior-predictive width attainment was 53.15%, compared with 0.03% when nearly the same budget retained the original validation sample. Increasing the precision valuation fourfold raised attainment to 10,000/10,000 simulations, with 95.31% coverage. Uncorrected specificity shifts produced substantial bias despite larger samples.

**Conclusions:**

At k = 3, the loss-based rule selected a screening-positive planning proportion of 0.1862, reducing conditional invitation requirements by 39.38% relative to p = 0.5. Joint survey–validation allocation improved simulated precision at comparable assumed cost. These findings support allocating recruitment and diagnostic-validation resources jointly when planning current-prevalence surveys.

## Introduction

Huang et al. [1] reported a weighted 12-month prevalence of major depressive disorder (MDD) of 2.1% (95% CI [1.8%, 2.4%]) among community-dwelling adults aged 18 years or older in the China Mental Health Survey, conducted in 2013–2015. A subsequent analysis documented substantial impairment and low treatment use among people with 12-month depressive disorders [2]. These findings motivate careful survey planning, but a 12-month disorder estimate does not determine the yield of a screener with a two-week symptom window. The national estimates therefore provide background only; none enters the numerical analysis.

For fixed precision, the usual sample-size formula for a proportion contains p(1−p) [3]. This term is maximised at p = 0.5 [3]. Thus, p = 0.5 gives the largest requirement over the full parameter space. The convention is appropriate when defensible prior information is unavailable or full-space protection is required. When expected positivity is low, however, it may increase recruitment without addressing misclassification, clustering or non-response. A lower planning value is defensible only when specified prospectively and evaluated under credible departures from its assumptions.

Bayesian sample-size methods have used posterior precision averaged over possible samples [4]. Binomial-proportion methods include average-coverage, average-length and worst-outcome criteria for highest posterior density intervals [5]. Generalised average-length and average-coverage criteria extend this framework [6]. Variance- and risk-based criteria have also been developed for conjugate binomial models [7]. Mixed Bayesian–likelihood criteria address comparisons of two proportions [8]. A decision-theoretic criterion instead assigns losses to the consequences of alternative sample sizes [9]. These approaches distinguish a precision requirement from a decision about the consequences of meeting or missing it.

Diagnostic uncertainty adds a variance contribution and an allocation decision. Bayesian prevalence planning has shown that uncertainty in misclassification rates can increase sample-size requirements and prevent a specified precision target from being reached [10]. Related methods address prevalence and diagnostic-test studies without a gold standard [11]. Testing strategies have also been compared by their accuracy and total cost [12]. The present application connects these established ideas to temporally aligned PHQ-9 evidence and clustered recruitment. It distinguishes conditional screening-loss decisions, decisions over the full invitation requirement, and allocation under a stated survey–validation cost-and-precision criterion.

The primary aim was to determine how aligned screening evidence and specified losses guide recruitment and diagnostic-validation allocation under uncertain diagnostic accuracy, clustering and response. A secondary aim was to quantify these decisions for two-week PHQ-9 positivity and current MINI-defined depression prevalence in an illustrative Chinese application, including comparisons with matched p = 0.5 designs.

## Methods

### Ethics approval and consent to participate

Not applicable. The study used published aggregate information and did not involve individual-level participant data.

### Estimands and measurement relation

Two outcomes were specified. Screening positivity was defined as PHQ-9 ≥ 7, using the instrument’s preceding-two-week symptom window [13]. The current reference outcome was defined by the MINI depression assessment used in the Chinese validation study [14]. With indicators S2w and Dcur for these outcomes, the parameters were p_s_ = P(S2w = 1), π = P(Dcur = 1), Se = P(S2w = 1 | Dcur = 1) and Sp = P(S2w = 0 | Dcur = 0). Screening positivity was calculated from the current-reference parameters by:


$$\begin{array}{c} p_{s}=P(\text{PHQ}\;-\;\text{9}\geq \text{7})=\text{Se}\;\pi +(\text{1}-\text{Sp})(\text{1}-\pi ). \end{array}$$ (1)

The source protocol administered the PHQ-9 followed by the MINI depression assessment, and described the MINI reference classification in relation to current major depressive episodes [14]. Screening planning used the directly observed PHQ-9-positive count. Current-prevalence calculations used the same study’s MINI-positive count and threshold-specific accuracy estimates, with p_s_ generated from Equation (1). S1 Appendix, Table S7 maps each empirical input to its outcome definition, source location and analytical use.

### Decision-theoretic selection of the planning screening-positive proportion

The planning parameter was the future survey’s two-week PHQ-9 screening-positive proportion p_s_. A Beta distribution with shape parameters α > 0 and β > 0 represented planning uncertainty:


$$\begin{array}{c} p_{s}\sim \text{Beta}(\alpha ,\beta ). \end{array}$$

Define $T=p_{s}\left(\text{1}-p_{s}\right)$, the component of binomial variance that depends on the proportion. Let $T^{*}$ ∈ [0, 0.25] be the value used for planning, $c_{o}$ the unit loss assigned to oversampling and $c_{u}$ the unit loss assigned to under-recruitment relative to the target half-width. Both unit losses are strictly positive. The asymmetric absolute loss is


$$\begin{array}{c} L\left(T^{*},T\right)=c_{o}{\left(T^{*}-T\right)}^{+}+c_{u}{\left(T-T^{*}\right)}^{+},\;\;(x{)}^{+}=\text{max}(x,\text{0}). \end{array}$$ (2)

The unrounded invitation requirement, with other inputs fixed, was proportional to T. The planning quantile was obtained by differentiating the expected loss in Equation (2): for candidate action a, the derivative was (c_o_ + c_u_)FT(a) − c_u_, where FT is the cumulative distribution function of T. Setting this derivative to zero gave:


$$\begin{array}{c} q=\frac{c_{u}}{c_{o}+c_{u}}=\frac{k}{\text{1}+k},\;\;k=\frac{c_{u}}{c_{o}}. \end{array}$$ (3)

The standard asymmetric linear-loss quantile rule was applied to the continuous variance term [15]. For t ∈ [0, 0.25], r(t) was defined as [1 − √(1 − 4t)]/2, and Fp_s_ denoted the Beta cumulative distribution function. The distribution of T was evaluated as:


$$\begin{array}{c} F_{T}(t)=F_{p_{s}}\{r(t)\}+\text{1}-F_{p_{s}}\{\text{1}-r(t)\}. \end{array}$$ (4)

Brent’s method solved $F_{T}\left(T^{*}\right)=q$. The two roots of T* = p_s_*(1 − p_s_*) give the same variance and invitation requirement. The lower root was selected for reporting in this low-positivity application:


$$\begin{array}{c} p_{s}^{*}=\frac{\text{1}-\sqrt{\text{1}-\text{4}T^{*}}}{\text{2}}. \end{array}$$ (5)

### Planning prior and updating

At PHQ-9 ≥ 7, the validation study reported 170 positive results among 1,045 residents of one Shanghai community [14]. These counts set the planning mean to μ_s_ = 170/1,045 = 0.162679. An investigator-specified effective sample size (ESS) of 100 discounted the source sample, giving α = 100μ_s_ = 16.2679426 and β = 100(1 − μ_s_) = 83.7320574. Full-precision parameters were used in all calculations.

For the local-update extension, the binomial likelihood for x screening-positive results among n independent pilot participants was combined with the planning prior:


$$\begin{array}{c} p_{s}|x\sim \text{Beta}(\alpha +x,\beta +n-x). \end{array}$$ (6)

### Loss-ratio specification

The base calculation specified an underplanning-to-overplanning marginal loss ratio k = 3, corresponding to q = 0.75. Loss-ratio sensitivity recalculated the quantile, planning proportion and invitation requirements at k = 1, 2, 3, 5, 10 and 20. These investigator-specified values were fixed before performance evaluation. A prospective elicitation template is provided in S1 Appendix, Section S10.

### Estimand-specific sample-size formulae

The anticipated mean analysed cluster size m̄, its coefficient of variation CVm and the screening-outcome intraclass correlation ρ defined the clustering adjustment. Under exchangeable outcomes and noninformative cluster sizes, the respondent-weighted factor 1 + ρ[Σ mc^2^/Σ mc − 1] was expressed using the cluster-size moments as:


$$\begin{array}{c} \text{DEFF}=\text{1}+\rho \left[\left(\text{1}+{\text{CV}}_{m}^{\text{2}}\right)\bar{m}-\text{1}\right]. \end{array}$$ (7)

Equation (7) used the unequal-cluster expression studied in cluster-randomised-trial planning [16]. Setting CVm = 0 gave the equal-cluster factor 1 + ρ(m̄ − 1) [17]. The other inputs were anticipated response RR, target 95% confidence-interval half-width d and z = 1.96. The candidate used p_s_,plan = p_s_*, and its comparator used p_s_,plan = 0.5. The final invitation requirement was rounded upward once using the ceiling operator.

For screening positivity $p_{s}$, the invitation requirement was calculated as:


$$\begin{array}{c} n_{\text{inv},s}=\text{ceil}\left\{\frac{z_{\text{0}.\text{9}\text{7}\text{5}}^{\text{2}}p_{s,\text{plan}}\left(\text{1}-p_{s,\text{plan}}\right)\;\text{DEFF}}{d^{\text{2}}\;\text{RR}}\right\}. \end{array}$$ (8)

For current reference prevalence, screening positivity was adjusted using the Rogan–Gladen estimator with fixed sensitivity and specificity [18]:


$$\begin{array}{c} \hat{\pi }=\frac{{\hat{p}}_{s}+\hat{\text{Sp}}-\text{1}}{\hat{\text{Se}}+\hat{\text{Sp}}-\text{1}}, \end{array}$$

where J=Se+Sp−1. Treating Se and Sp as fixed gives


$$\begin{array}{c} n_{\text{inv},\pi }^{\left(\text{fixed}\right)}=\text{ceil}\left\{\frac{z_{\text{0}.\text{9}\text{7}\text{5}}^{\text{2}}p_{s,\text{plan}}\left(\text{1}-p_{s,\text{plan}}\right)\;\text{DEFF}}{d^{\text{2}}\;\text{RR}\;J^{\text{2}}}\right\}. \end{array}$$ (9)

The conditional current-prevalence candidate substituted p_s_* from the screening-loss calculation into Equation (9). Its performance was then evaluated under a joint distribution of current prevalence and diagnostic accuracy, generating p_s_ from Equation (1) for each draw. Both estimands used d = 0.020 and untruncated normal-approximation intervals. The fixed-accuracy reference-point comparison used p_s_,plan = p_s,0_.

### Diagnostic-accuracy uncertainty and the precision floor

The diagnostic-variance calculation was evaluated at a coherent reference point: p_s,0_ = Seπ_0_ + (1 − Sp)(1 − π_0_). The delta method was applied to g(p_s_, Se, Sp) = (p_s_ + Sp − 1)/(Se + Sp − 1), using gradient (1, − π_0_, 1 − π_0_)/J. The survey, validation and cross-sample components were calculated as:


$$\begin{array}{c} V_{s}=\text{Var}\left({\hat{p}}_{s}\right)\approx \frac{p_{s,\text{0}}\left(\text{1}-p_{s,\text{0}}\right)\;\text{DEFF}}{n_{\text{inv}}\text{RR}}, \end{array}$$

$$\begin{array}{c} V_{D}\left(\pi_{\text{0}}\right)=\pi_{\text{0}}^{\text{2}}\text{Var}\left(\hat{\text{Se}}\right)+{\left(\text{1}-\pi_{\text{0}}\right)}^{\text{2}}\text{Var}\left(\hat{\text{Sp}}\right)-\text{2}\pi_{\text{0}}\left(\text{1}-\pi_{\text{0}}\right)\text{Cov}\left(\hat{\text{Se}},\hat{\text{Sp}}\right), \end{array}$$

and


$$\begin{array}{c} V_{SV}\left(\pi_{\text{0}}\right)=-\text{2}\pi_{\text{0}}\text{Cov}\left({\hat{p}}_{s},\hat{\text{Se}}\right)+\text{2}\left(\text{1}-\pi_{\text{0}}\right)\text{Cov}\left({\hat{p}}_{s},\hat{\text{Sp}}\right). \end{array}$$

Then


$$\begin{array}{c} \text{Var}\left(\hat{\pi }\right)\approx J^{-\text{2}}\{V_{s}+V_{D}\left(\pi_{\text{0}}\right)+V_{SV}\left(\pi_{\text{0}}\right)\}. \end{array}$$ (10)

The reference scenario used π_0_ = 28/1,045 and rounded Se = Sp = 0.86 from the Shanghai study [14]. Equation (1) then gave p_s,0_ = 0.1592919. All variance components were evaluated at this point. Validation was modelled as an external sample independent of the survey, with independent-binomial accuracy estimates conditional on fixed reference-positive and reference-negative denominators n_1_ and n_0_. Accordingly, VSV and Cov(Sê, Sp̂) were set to zero, giving:


$$\begin{array}{c} V_{D}\left(\pi_{\text{0}}\right)=\pi_{\text{0}}^{\text{2}}\frac{\text{Se}(\text{1}-\text{Se})}{n_{\text{1}}}+{\left(\text{1}-\pi_{\text{0}}\right)}^{\text{2}}\frac{\text{Sp}(\text{1}-\text{Sp})}{n_{\text{0}}}. \end{array}$$

For each fixed independent-validation allocation, the main-survey requirement was calculated as:


$$\begin{array}{c} n_{\text{inv},\pi }^{\left(\text{ext}\right)}=\text{ceil}\left\{\frac{p_{s,\text{0}}\left(\text{1}-p_{s,\text{0}}\right)\;\text{DEFF}}{\text{RR}\left[d^{\text{2}}J^{\text{2}}/z_{\text{0}.\text{9}\text{7}\text{5}}^{\text{2}}-V_{D}\left(\pi_{\text{0}}\right)\right]}\right\}. \end{array}$$ (11)

For J > 0 and 0 < p_s,0_ < 1, the available survey-variance budget was calculated as B = d^2^J^2^/z^2^ − VD(π_0_). Equation (11) was evaluated when B > 0; otherwise, the requirement was recorded as infinite. The limiting first-order half-width was calculated as z√VD(π_0_)/J.

The fixed-accuracy lower bound was checked by evaluating Equations (9) and (11) at the same p_s,0_, d, DEFF, RR, Se and Sp. At VD(π_0_) = 0 the formulae coincide. For 0 < VD(π_0_) < d^2^J^2^/z^2^, the positive validation term reduces the survey-variance budget and increases the unrounded requirement; upward rounding preserves the ordering. At or above this boundary, the positive survey-variance component prevents a finite solution. This comparison used the coherent reference point in both formulae.

### Sampling and validation assumptions

The base calculations used exchangeable screening outcomes, noninformative cluster sizes, outcome-independent response and diagnostic accuracy defined for the target screening and reference classifications. Covariance terms were retained in Equation (10) and set to zero for the independent external-validation calculations. Independence between prior marginals was specified separately for the base integration analyses.

### Illustrative Chinese depression-surveillance application

The base case specified d = 0.020, Se = Sp = 0.86, ρ = 0.03, m̄ = 50, CV_m_ = 0, RR = 0.75, ESS = 100 and k = 3. The PHQ-9 threshold, positivity count, validation denominators and rounded diagnostic-accuracy estimates came from the Shanghai study [14]. Precision, clustering, response, prior strength, losses and sensitivity distributions were investigator-selected inputs. The comparator changed only p_s_,plan to 0.5. Percentage difference was calculated as 100 × [1 − n(p_s_*)/n(0.5)].

### Sensitivity analyses

ESS sensitivity used 5, 10, 25, 50, 100 and 200 while holding the planning mean at 170/1,045. Prior-location sensitivity used means 0.10, 0.13, 170/1,045, 0.20 and 0.25 at ESS 100. The alternative logit-normal distributions fixed logit-location at logit(170/1,045), giving probability-scale median 170/1,045, and used logit-scales τ = 0.05, 0.15, 0.30, 0.50 and 0.75. Each specified distribution was transformed to T, and the same quantile rule and sample-size formulae were applied.

Specificity sensitivity used Sp = 0.86, 0.84, 0.82, 0.80 and 0.78 at Se = 0.86 and π_0_ = 28/1,045. Each scenario recalculated the model-implied screening mean, its ESS-100 Beta distribution, the planning quantile and J^-2^. This scenario series used the rounded-accuracy model mean, whereas the primary prior used observed positivity. Validation sensitivity retained n_1_ = 28 and varied n_0_ over 1,017, 2,500, 5,000 and 10,000. Analytical boundaries varied one parameter at a time while holding the other formula inputs fixed.

The source-consistency check enumerated integer diagnostic tables compatible with the published margins and sensitivity and specificity rounded to two decimals. Predictive values and likelihood ratios calculated from each compatible table were compared with the published summaries. The rounding analysis evaluated the four corners of [0.855, 0.865]^2^. It held the observed-positivity prior, p_s_*, π_0_ and survey-design inputs fixed, recalculated J and coherent p_s,0_, and recomputed candidate invitations and the validation floor.

### Joint parameter assurance

For estimand e, let n_e_(k) be the candidate invitation count and N_req,e_(θ) the formula-based requirement at planning parameters θ. Parameter assurance was defined as:


$$\begin{array}{c} A_{e}(k)=P_{\theta }\{N_{\text{req},e}\left(\theta \right)\leq n_{e}(k)\}. \end{array}$$ (12)

For each candidate, Equation (12) was estimated as the fraction of parameter draws whose formula-based requirement did not exceed the candidate invitation count. The resulting parameter assurance was compared with a prespecified 0.90 benchmark. Predictive interval-width attainment was evaluated separately by generating future survey and validation data.

For screening positivity, the specified marginals were p_s_ ~ Beta(100 × 170/1,045, 100 × 875/1,045), ρ ~ Triangular(0.02, 0.03, 0.05) and RR ~ Triangular(0.60, 0.75, 0.85). For current reference prevalence, they were π ~ Triangular(0.01, 28/1,045, 0.10), Se ~ Triangular(0.78, 0.86, 0.90) and Sp ~ Triangular(0.80, 0.86, 0.90), with the same ρ and RR distributions. Triangular arguments denote minimum, mode and maximum. Each current-prevalence draw generated screening positivity through Equation (1).

Independent marginals defined the base joint distribution. A PCG64 stream with seed 20260722 generated 1,000,000 draws per estimand, with screening draws generated first. Each draw’s Se and Sp values were inserted into the fixed-accuracy requirement in Equation (9). The resulting requirements were used to evaluate conditional candidates and calculate joint recruitment-loss decisions.

### Bayes decisions under joint uncertainty

At each joint-distribution draw θ, Equation (8) or (9) was rounded upward to obtain the integer requirement R_e_(θ). The loss was specified as c_o_(n − R_e_)₊ + c_u_(R_e_ − n)₊. Under finite E(R_e_), its Bayes action was calculated as the smallest integer reaching the q-quantile:


$$\begin{array}{c} n_{q}\;=\;min\{n\;\in \;N\;:\;F_{R_{e}}(n)\;\geq \;q\},\;q\;=\;\frac{k}{\text{1}+k}. \end{array}$$ (13)

Equation (13) was evaluated for the same six k values and for the 0.90 assurance benchmark, corresponding to k = 9. The empirical inverse CDF of the 1,000,000 integer requirements was calculated without interpolation. S1 Appendix gives the integer-loss derivation and the finite-risk conditions.

### Joint allocation of survey and validation samples

The current-prevalence allocation action was x = (C, n_1_, n_0_), with integers C, n_1_, n_0_ ≥ 2. C denoted survey clusters, and n_1_ and n_0_ denoted independent reference-positive and reference-negative validation counts. Each cluster received b = 67 invitations. Conditional on θ = (π, Se, Sp, ρ, RR), the first-order ratio-estimator variance under Bernoulli response and exchangeable screening outcomes was calculated as:


$$\begin{array}{c} V(x;\;\theta )\;=\;\frac{a_{C}(\theta )}{C}\;+\;\frac{a_{\text{1}}(\theta )}{n_{\text{1}}}\;+\;\frac{a_{\text{0}}(\theta )}{n_{\text{0}}}. \end{array}$$ (14)

The coefficients were aC = p_s_(1 − p_s_)[1 + (b − 1)RRρ]/(b RR J^2^), a_1_ = π^2^Se(1 − Se)/J^2^ and a_0_ = (1 − π)^2^Sp(1 − Sp)/J^2^. Screening positivity was calculated using Equation (1). The survey coefficient used binomial respondent counts at fixed b; the validation coefficients used conditional binomial sampling variances. Coefficient expectations were integrated over the previously specified current-prevalence joint prior.

The allocation loss was specified as total cost plus λ times first-order estimation variance. With positive unit costs c = (cC, c_1_, c_0_), λ > 0 and ā_j_ = Eθ[a_j_(θ)], the Bayes risk was:


$$\begin{array}{c} R(x)=\Sigma_{j}c_{j}x_{j}+\lambda \Sigma_{j}\frac{{\overset{―}{a}}_{j}}{x_{j}},\;x=(C,n_{\text{1}},n_{\text{0}}). \end{array}$$ (15)

For each component, the continuous minimiser x_j_* = √(λā_j_/c_j_) was calculated. Its floor and ceiling were evaluated after applying the lower bound xⱼ ≥ 2, and the lower-risk integer was selected; ties selected the smaller count. S1 Appendix provides the convexity proof, the continuous fixed-budget allocation and the numerical optimality checks.

Illustrative all-inclusive costs were set to one relative unit per invitation and five per completed validation participant, giving c = (67, 5, 5). The base precision valuation was calibrated as λ_0_ = [Σ_j_√(c_j_ā_j_)/v_0_]^2^, where v_0_ = (d/z)^2^, setting the continuous allocation’s prior mean variance to v_0_. Coefficient expectations used Gauss–Legendre quadrature split at triangular modes, with convergence checked at 32 and 48 nodes per segment. The integer allocation’s expected variance was recalculated after optimisation.

Allocation sensitivity used λ/λ_0_ = 0.25, 0.5, 1, 2 and 4. Cost sensitivity used (c_1_, c_0_) = (1,1), (5,5), (20,5) and (5,20), holding λ_0_ and invitation cost fixed. Prior sensitivity preserved triangular marginals while separately setting latent Gaussian-copula correlation to −0.5 for Se/Sp or ρ/RR. A wider-accuracy scenario used Se ~ Triangular(0.60,0.86,0.95) and Sp ~ Triangular(0.75,0.86,0.95). Each scenario used 1,000,000 parameter draws. The reoptimised allocation and the unchanged base design were evaluated under each applicable prior scenario.

### Cluster simulation operating characteristics

Cluster simulation evaluated three planning values for current reference-standard prevalence: p_s_* = 0.1862346, p_s_ = 0.30 and p_s_ = 0.50. Their fixed-accuracy invited samples were 9,247, 12,813 and 15,254. For each strategy, C = ceil(ninv × 0.75/50) gave 139, 193 and 229 clusters. Invitations were divided as evenly as possible. One additional invitation was assigned to each of the first remainder clusters. The cluster counts and invitation allocations were held fixed across scenarios.

Cluster screening-positive risks followed Beta(a,b), where a = p_s_(ρ^-1^−1) and b = (1 − p_s_)(ρ^-1^−1). Respondent counts were binomial with the scenario response probability. Screening-positive counts were binomial conditional on respondent counts and cluster risk.

Let Yc and Nc denote screening-positive and respondent counts in cluster c. For C clusters, the screening variance estimate was [C/(C − 1)]Σc(Yc − p̂_s_ Nc)^2^/(Σc Nc)^2^ The untruncated Rogan-Gladen estimate and normal interval used either true scenario-specific accuracy or unrecalibrated Se = Sp = 0.86. Width attainment was the fraction of replicates with half-width ≤0.020 using true scenario accuracy. Coverage was reported separately for both analyses.

Departures comprised π = 0.10, Sp = 0.80, Se = 0.78, or the joint change ρ = 0.05 and RR = 0.60. The combined adverse scenario used π = 0.05, Se = Sp = 0.80, ρ = 0.05 and RR = 0.60.

Informative response used logit(RRc) = ar − 0.75 zc. Within each replicate, zc was centred and scaled by the sample mean and standard deviation of cluster risk; ar was chosen to give an invitation-weighted response probability of 0.75. Each of the 21 scenario-strategy cells used 10,000 replicates and a separate child stream from seed 20260722.

### Finite-validation and transportability simulations

The finite-validation simulations evaluated the integer allocations at λ_0_ and 4λ_0_ and a comparator retaining n_1_ = 28 and n_0_ = 1,017. The comparator allocated the remaining base-design budget to complete 67-invitation clusters. For each replicate, cluster screening risks were beta-distributed with mean p_s_ and ICC ρ. Respondent counts followed Binomial(67, RR), and screening-positive counts were binomial conditional on respondents and cluster risk. Independent true-positive and true-negative validation counts followed Binomial(n_1_, Seval) and Binomial(n_0_, Spval). The Rogan–Gladen estimate used the resulting estimated accuracy. Its untruncated interval combined the cluster variance estimate with the two plug-in validation components from Equation (14).

Each of 15 design–scenario cells used 10,000 replicates. Scenarios were the reference point; draws of all five parameters from the base joint prior; target Sp = 0.80 with source validation Seval = Spval = 0.86; the same target with local validation Spval = 0.80; and target Se = 0.78 with source validation retained at 0.86. Non-positive estimated J or an undefined interval counted as both width and coverage failure. Outcomes were width attainment, coverage, their joint occurrence, bias and mean squared error. The prior-predictive scenario integrated parameter and future-data uncertainty; the others evaluated fixed departures.

An analytical transportability grid additionally crossed ΔSe = 0, −0.04, −0.08 with ΔSp = 0, −0.02, −0.06 at π_0_, relative to source accuracy 0.86. The limiting bias when source accuracy is retained is [π_0_ΔSe − (1 − π_0_)ΔSp]/Jsource. S1 Appendix gives the complete grid and simulation recipe. New random streams used SeedSequence([20260722, 20260907, s]); s = 1 for base allocation draws, 2–4 for prior sensitivity, and 19 with 15 spawned child streams for future-data simulations.

The analysis used Python 3.12.13, NumPy 2.3.5, pandas 2.2.3, SciPy 1.17.0 and Matplotlib 3.10.8. Independent checks covered loss integration, a uniform-prior closed form, numerical derivatives, variance budgets, quadrature convergence and integer optimality. Monte Carlo standard errors and Wilson intervals were reported for simulated proportions, including zero-event cells. S1 Appendix provides proofs, exact simulation settings and the output-file map.

## Results

### Planning value and base-case sample sizes

At k = 3, the conditional loss rule selected T* = 0.1515513 and the lower-root planning value p_s_* = 0.1862346. This value is neither the prior mean 0.1627 nor an estimate of disorder prevalence. With design effect 2.47, screening positivity required 4,794 invitations, compared with 7,908 under p = 0.5.

Current reference-standard prevalence required 9,247 invitations when Se = Sp = 0.86 were treated as known, compared with 15,254 under matched p = 0.5 planning (Table 1). Both conditional differences were 39.38%. These comparisons hold all design inputs fixed.

**Table 1:** Base-case invited sample size by estimand and planning proportion.

Estimand	Planning input	Invitations	Difference from p = 0.5
PHQ-9 positivity	p_s_* = 0.1862	4,794	39.38% smaller
PHQ-9 positivity	p = 0.5000	7,908	Reference
Current reference prevalence	p_s_* = 0.1862	9,247	39.38% smaller
Current reference prevalence	p = 0.5000	15,254	Reference

Note. All calculations use d = 0.02, ρ = 0.03, a mean of 50 analysed respondents per cluster, cluster-size CV = 0 and RR = 0.75. The current-prevalence rows treat Se = Sp = 0.86 as fixed. The same numerical d is illustrative and does not make the two estimands substantively equivalent.

### Recruitment under parameter uncertainty

Increasing the relative loss for under-recruitment increased the planning value and conditional sample requirement (Table 2). Across k ∈ {1, 2, 3, 5, 10, 20}, p_s_* ranged from 0.1604 to 0.2278. The screening-positive requirement ranged from 4,261–5,564. The fixed-accuracy current-prevalence requirement ranged from 8,218–10,732. Relative to p = 0.5, the corresponding conditional differences decreased from 46.12% to 29.64% for screening positivity. They decreased from 46.13% to 29.64% for current reference-standard depression. No empirical cost evidence distinguished k = 3 from the other prespecified values.

**Table 2:** Candidate loss-ratio designs and joint parameter assurance.

k (q)	p_s_*	Screening invitations	Screening parameter assurance	Current-prevalence invitations	Current-prevalence parameter assurance
1 (0.5000)	0.1604	4,261	37.24%	8,218	16.43%
2 (0.6667)	0.1767	4,601	50.22%	8,876	26.59%
3 (0.7500)	0.1862	4,794	57.37%	9,247	32.98%
5 (0.8333)	0.1981	5,024	65.34%	9,691	40.88%
10 (0.9091)	0.2134	5,310	74.02%	10,242	50.65%
20 (0.9524)	0.2278	5,564	80.47%	10,732	58.86%

Note. Parameter assurance evaluates the formula-based requirement under the stated independent marginal distributions; it does not integrate future data. Current-prevalence draws derive p_s_ from π, Se and Sp and treat those accuracy values as known. With 1,000,000 draws, the maximum Monte Carlo standard error is 0.0005. Finite validation variance is assessed separately.

Under the specified joint distributions, parameter assurance increased with k but remained below 0.90 for all six candidates (Table 2). Screening assurance ranged from 37.24% to 80.47%; current-prevalence assurance ranged from 16.43% to 58.86%. At k = 3, the corresponding values were 57.37% and 32.98%. These are probabilities that a formula-based requirement fits the candidate size under parameter uncertainty, not the base-scenario simulated width-attainment probability of 84.11%.

Applying the same k = 3 recruitment loss to the full uncertain requirement selected 5,346 screening invitations and 11,866 oracle-accuracy current-prevalence invitations. The corresponding parameter assurances were 75.01% for both estimands, compared with 57.37% and 32.98% for the original conditional candidates. The declared 90% benchmark required 6,089 and 13,551 invitations. These values use the complete requirement distribution rather than a variance quantile multiplied by fixed design factors.

### Prior sensitivity

Prior strength affected the calculation. At mean 170/1,045 and k = 3, reducing ESS from 100 to 5 increased p_s_* from 0.1862 to 0.2389. The fixed-accuracy current-prevalence sample increased from 9,247–11,094. At ESS 200, p_s_* = 0.1796 and the sample was 8,992 (Fig 1A). Varying the prior mean from 0.10 to 0.25 at ESS 100 changed the sample from 6,376–12,254. Sensitivity under the logit-normal model also depended on τ. Increasing τ from 0.05 to 0.30 raised p_s_* from 0.1673 to 0.1922. The sample increased from 8,501–9,472. At τ = 0.75, p_s_* = 0.2436 and the sample was 11,244. These results confirm that the prescribed distribution is a discounted planning input rather than a posterior identified by one study.

**Fig 1:**
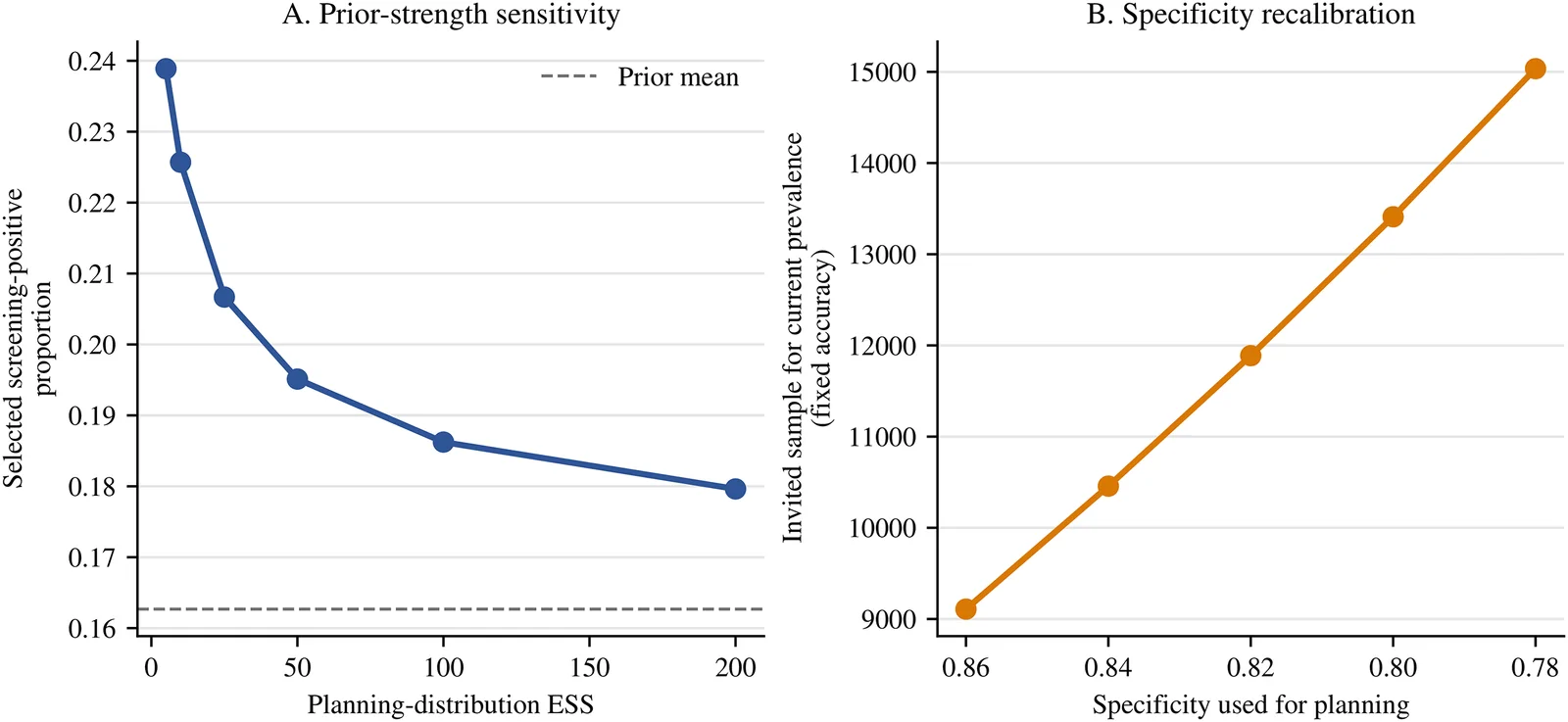
Sensitivity to planning-distribution ESS and specificity. Panel A holds the prior mean at the directly observed PHQ-9-positive proportion 170/1,045 and k = 3. Panel B reconstructs screening positivity from current reference-standard prevalence and recalculates both the prior location and diagnostic inflation as specificity changes, with sensitivity fixed at 0.86.

### Diagnostic accuracy, transportability and validation precision

Specificity affected both model-implied screening positivity and variance inflation (Table 3; Fig 1B). At Sp = 0.86 and π_0_ = 28/1,045, the reconstructed mean was 0.1593 and the candidate requirement was 9,108. The principal observed-positivity prior instead gave 9,247. Reducing specificity to 0.80 increased the reconstructed mean to 0.2177, p_s_* to 0.2444 and the requirement to 13,411. At Sp = 0.78, the requirement was 15,035, compared with 19,305 under the matched p = 0.5 design. This 22.12% difference holds diagnostic accuracy constant between strategies.

**Table 3:** Specificity-recalibrated planning values.

Planning Sp	Model-implied prior mean p_s_	Selected p_s_*	Fixed-accuracy invitations
0.86	0.1593	0.1826	9,108
0.84	0.1788	0.2033	10,457
0.82	0.1982	0.2239	11,888
0.80	0.2177	0.2444	13,411
0.78	0.2371	0.2648	15,035

At the coherent reference point π₀ = 28/1,045 and p_s,0_ = 0.1592919, the independent-binomial validation model imposed a minimum 95% half-width of 0.0292199 (Fig 2). The finite-validation precision budget was therefore negative at d = 0.020, so Equation (11) had no finite solution. Specificity contributed 97.32% of diagnostic variance. Increasing only n_0_ to 2,500 reduced the floor to 0.0189973 and required 83,583 main-survey invitations. Values n_0_ = 5,000 and 10,000 required 15,705 and 11,170 invitations. These are pointwise reference-scenario requirements, rather than the loss-based candidate sizes in Table 1; the complete calculation is supplied in validation_precision.csv.

**Fig 2:**
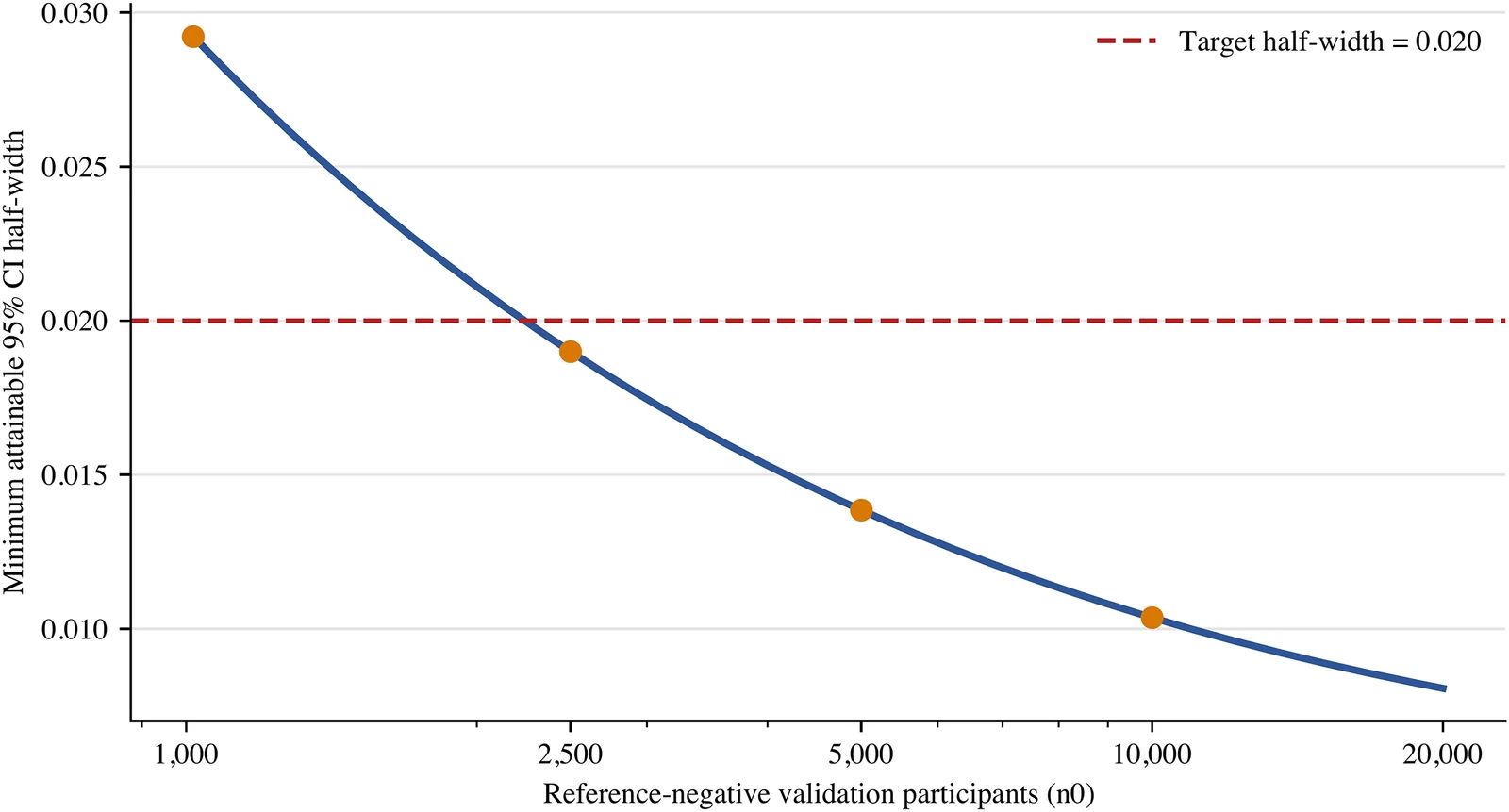
Validation sample size and the precision limit for current prevalence. The curve shows the minimum attainable 95% confidence-interval half-width for current reference-standard prevalence as the reference-negative validation sample increases. The reference-positive validation sample remains fixed at 28. The horizontal line is the prespecified 0.020 target; π₀ = 28/1,045 and Se = Sp = 0.86.

The source reports a positive predictive value of 0.15 at PHQ-9 ≥ 7 [14]. Under one unweighted diagnostic table with the published margins, sensitivity rounding to 0.86 implies 24 true positives, giving 24/170 = 0.1412, which rounds to 0.14. The published summaries therefore did not establish an exact internally consistent diagnostic table. Predictive values were excluded from the planning inputs. Across the diagnostic-rounding enclosure, fixed-accuracy candidate invitations ranged from 8,996–9,509, and the coherent validation floor ranged from 0.02838 to 0.03007. The 0.020 target remained unattainable with the fixed validation sample throughout this enclosure.

### Simulated width attainment, coverage and failure boundaries

The 9,247-invitation candidate attained the 0.020 interval-width target in 84.11% of base-scenario replicates (Table 4). Width attainment was 22.94% at π = 0.10, 1.55% at Sp = 0.80 and 23.98% at Se = 0.78. It was 2.75% under the adverse ICC-response scenario. The p = 0.50 design attained 98.31%−100% across these single-departure scenarios. Under the combined adverse condition, all three strategies recorded 0 successes in 10,000 replicates. These empirical zeros do not establish a zero underlying probability.

**Table 4:** Simulated width attainment and coverage under parameter misspecification.

Scenario	Width attainment: p_s_*	Width attainment: p = 0.30	Width attainment: p = 0.50	Base-accuracy coverage: p_s_*
Base case	0.8411	1.0000	1.0000	0.9480
π = 0.10	0.2294	0.9889	1.0000	0.9507
Sp = 0.80	0.0155	0.6473	0.9861	0.0000
Se = 0.78	0.2398	0.9807	1.0000	0.9300
ρ = 0.05; RR = 0.60	0.0275	0.6661	0.9831	0.9462
Informative response	0.9958	1.0000	1.0000	0.5343
Combined adverse	0.0000	0.0000	0.0000	0.0000

Note. Width attainment is the fraction of 10,000 replicates with untruncated 95% half-width ≤0.020 using scenario-specific accuracy. The final column uses the unrecalibrated base accuracy. A displayed zero represents 0/10,000, not a proven zero probability; its two-sided 95% Wilson upper bound is approximately 0.000384. The maximum Monte Carlo standard error is 0.005. Finite validation uncertainty is excluded.

With Sp = 0.80, the candidate’s coverage was 94.79% when the analysis used the correct scenario accuracy, but 0/10,000 when it retained Se = Sp = 0.86. Under informative response, width attainment was at least 99.58%, yet coverage was 53.43%, 40.80% and 35.30% for the three strategies (Fig 3). Thus, a narrow interval could coexist with substantial selection bias. In the correctly specified, noninformative candidate scenarios, true-accuracy coverage ranged from 94.42% to 95.07%; full results and Monte Carlo intervals are provided in the simulation output.

**Fig 3:**
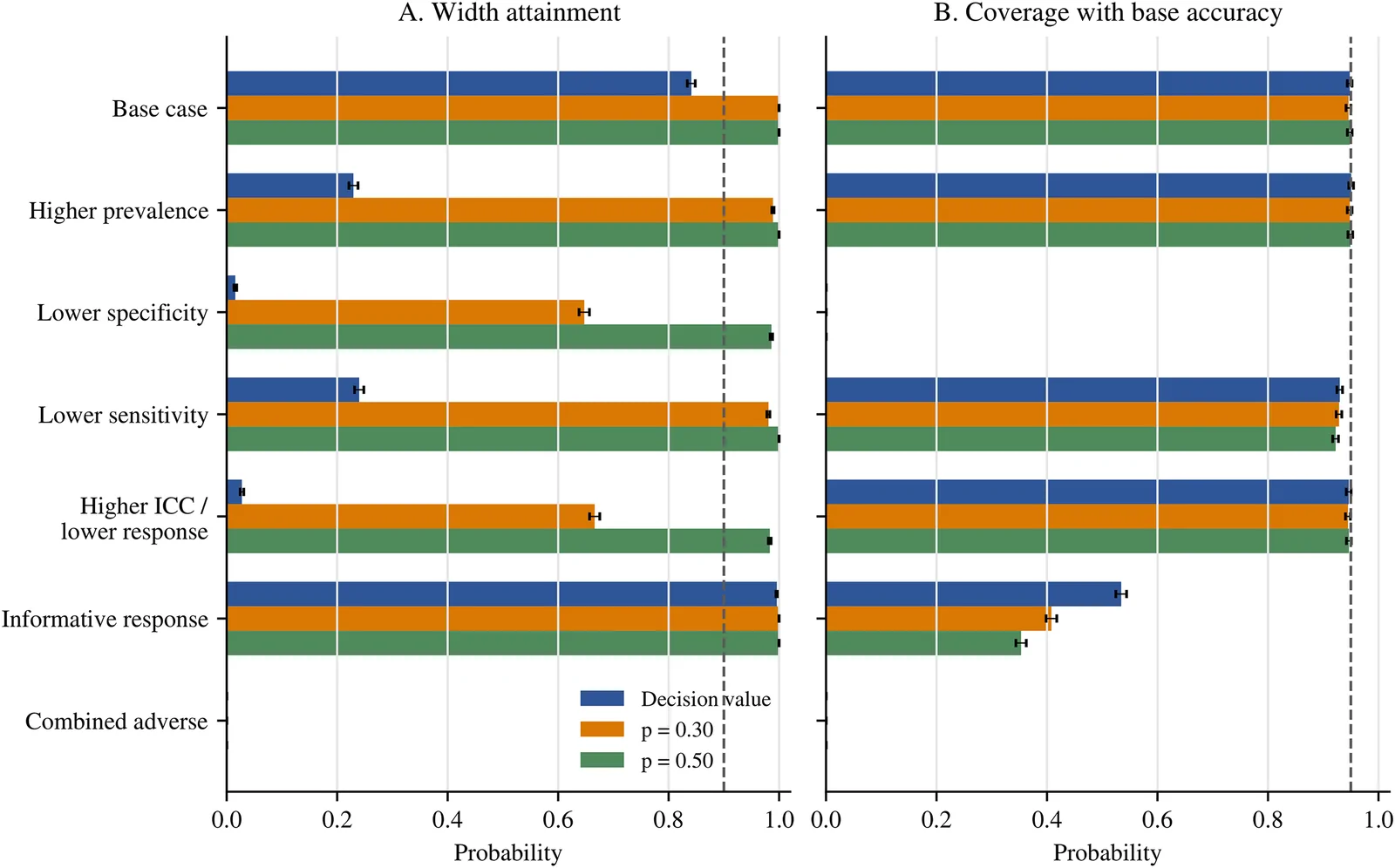
Cluster-simulation operating characteristics. Panel A shows interval-width attainment using scenario-specific diagnostic accuracy; its dashed line marks 90%. Panel B shows coverage when the analysis retains Se = Sp = 0.86; its dashed line marks 95%. Each cell uses 10,000 replicates. Bars include 95% Monte Carlo Wilson intervals.

Under the fixed formula inputs, the 9,247-invitation design met the pointwise target on the low-positivity branch while p_s_ ≤ 0.186245, equivalent to π ≤ 0.064229. At π = 28/1,045, the corresponding largest ICC was 0.036640 and smallest response rate was 0.662708.

These boundaries hold analysed mean cluster size at 50. The simulation instead fixes cluster count, allowing response changes to alter realised cluster size; the two calculations therefore address different allocation assumptions.

The original validation sample imposed a precision constraint that no change in k could remove at the reference point. The extended allocation analysis therefore treated validation capacity as a design variable. The original loss-ratio graphic is retained as Fig S1 in S1 Appendix.

### Joint allocation and future-data performance

Under the joint cost-plus-variance criterion, the base optimum was 324 clusters, 21,708 invitations, 240 reference-positive and 4,538 reference-negative validation participants. Its cost was 45,598 relative units. The rounded optimum had 1.96√E(V) = 0.020003 and parameter assurance of 53.72%; prior-predictive width attainment was 53.15% and coverage was 95.05%. Retaining the original validation counts at nearly the same cost (45,559 units) allowed 40,334 invitations but produced 0.03% predictive width attainment and 95.44% coverage (Table 5).

**Table 5:** Joint allocation and prior-predictive performance.

Design	Invitations	Validation n_1_ / n_0_	Relative cost	Width attainment	Coverage
Joint, λ_0_	21,708	240 / 4,538	45,598	0.5315	0.9505
Joint, 4λ_0_	43,416	481 / 9,077	91,206	1.0000	0.9531
Fixed validation, matched budget	40,334	28 / 1,017	45,559	0.0003	0.9544

Note. Width attainment and coverage integrate the stated parameter prior and future survey/validation data, with 10,000 replicates per row. Costs use one unit per invitation and five per completed validation participant. The matched-budget comparator costs 39 units less because clusters are indivisible. Values 0.0003 and 1.0000 represent 3 and 10,000 successes. These are different designs from the oracle-accuracy calculations in Tables 1–4.

At 4λ_0_, the optimum used 648 clusters, 43,416 invitations and validation counts of 481/9,077, costing 91,206 units. Width attainment was 10,000/10,000 and coverage was 95.31% in the prior-predictive simulation. The two-sided 95% Wilson lower bound for the universal width-success count was 99.96%, so the simulation does not establish a true success probability of one. Fig 4 contrasts joint allocation with spending on the survey while keeping validation fixed.

**Fig 4:**
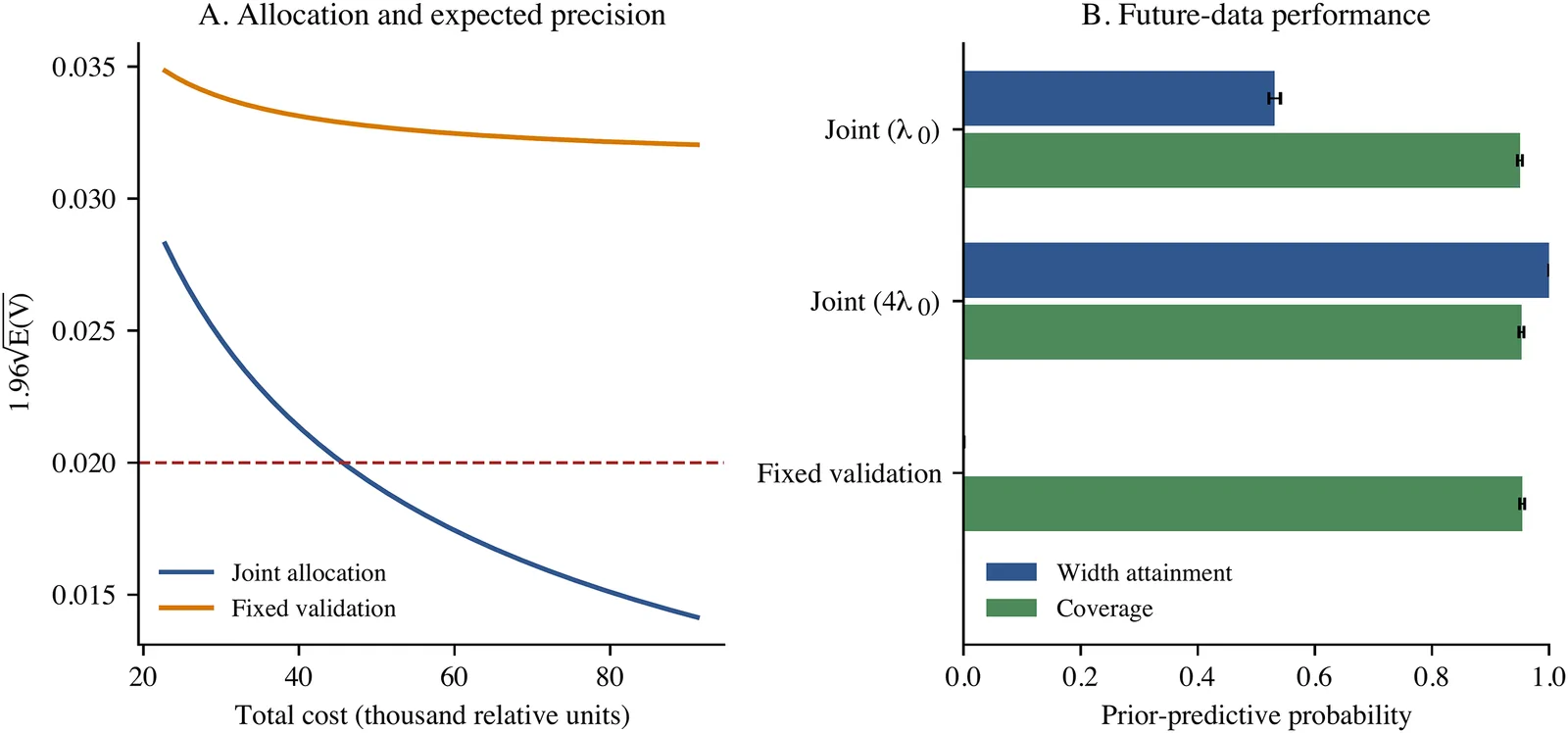
Joint survey–validation allocation and precision. Panel A compares the continuous minimum prior mean variance at each assumed budget with retaining 28/1,017 validation participants. The plotted quantity is 1.96√E(V), not an expected realised interval width. Panel B reports prior-predictive width attainment and coverage for the three designs in Table 5; bars show 95% Monte Carlo Wilson intervals. Costs are illustrative relative units.

Accuracy shifts affected validity as well as precision. With target specificity 0.80 and source validation retained at 0.86, limiting bias was + 0.0811 at π_0_. At 4λ_0_, simulated width attainment remained 100%, but coverage was 0/10,000. Local validation at target specificity restored coverage to 94.83%. Wider accuracy priors changed the reoptimised counts to 23,718 invitations and 294/4,993 validation participants; the unchanged base design’s parameter assurance fell to 40.54%. Full cost, dependence and transportability results are supplied in S1 Appendix and the numerical output.

## Discussion

The calculations show how recruitment decisions change when uncertainty is integrated into the decision itself. At k = 3, the conditional screening-loss rule produced 4,794 screening invitations, whereas loss on the full requirement produced 5,346 after integrating clustering and response. The corresponding current-prevalence values were 9,247 and 11,866 under oracle diagnostic accuracy. Thus, the 39.38% difference from conditional p = 0.5 calculations describes a fixed-input comparison; it does not quantify the gain from a complete uncertain-input design.

The decision-theoretic contribution is the explicit separation of objectives and action spaces. The quantile rule is established decision theory [15]. Applying it to the entire invitation requirement gives a Bayes recruitment decision under the specified joint distribution. It cannot assign costs to validation, and an infinite requirement with positive prior probability makes its expected shortfall loss infinite. The cost-plus-variance criterion supplies a finite, explicitly defined alternative that allocates survey clusters and both validation strata jointly. Its global optimum is proved for the stated first-order risk, rather than asserted for every design objective.

Validation allocation had a larger effect than adding main-survey invitations in the evaluated budget comparison. At relative costs of 45,598 and 45,559 units, joint allocation achieved 53.15% prior-predictive width attainment, whereas retaining the original validation sample achieved 0.03% despite 40,334 invitations. This result follows from the three separate variance components: more survey clusters reduce only the survey component. The reference-point specificity contribution of 97.32% also depends on the validation allocation. With both strata free to vary, the optimum balances marginal precision per cost unit; it does not imply that specificity is universally the only validation priority.

The allocation model uses independent external validation, a setting for which adjusted-prevalence intervals have been studied [19]. Validation nested within the main survey introduces a different sampling problem because phase-two selection enters prevalence estimation [20]. The budget comparison therefore addresses the external-validation design specified here.

A Bayes optimum remains specific to its utility. The base allocation was calibrated to a continuous-design prior mean variance corresponding to d = 0.020, yet its predictive width attainment was 53.15%. Increasing λ to 4λ_0_ produced 10,000/10,000 width successes and 95.31% coverage under the stated prior-predictive model. This improvement required approximately twice the assumed total cost. The comparison does not identify an empirically correct λ or k; a probability-of-success requirement should be declared as a separate design constraint when that is the commissioning objective.

Diagnostic transportability changes the interpretation of additional precision. When target specificity fell from 0.86 to 0.80 but validation retained source accuracy, the large-precision-value design had zero coverage in 10,000 simulations despite universal width attainment. Local validation at target specificity restored coverage to 94.83% in that design. Published PHQ-9 analyses have also documented sensitivity bias associated with selective cutoff reporting [21]. These findings support validating the chosen threshold in the intended population before treating a narrower interval as improved inference. S1 Appendix retains the practical decision rules for field implementation.

The informative-response simulation identified a separate failure mechanism: width attainment exceeded 99% while coverage fell to 35.30%–53.43%. Increasing sample size did not correct selection into the responding sample. This finding limits the interpretation of recruitment inflation and supports incorporating response mechanisms into survey analysis and subsequent design evaluation.

### Limitations

The empirical basis limits transportability. One Shanghai community sample supplies the screening and validation inputs; discounting its effective sample size does not establish national representativeness. Accuracy against current MINI classification concerns an observed reference standard rather than error-free MDD. The source describes the order of PHQ-9 and MINI administration but does not report their exact elapsed interval. Neither analysed outcome estimates 12-month prevalence. Inconsistencies among the published diagnostic summaries prevent confirmation of an exact validation table. The numerical rounding range cannot provide a sampling confidence interval or quantify between-population accuracy variation.

The design priors, cost ratios, k and λ were investigator-specified. No local pilot, stakeholder valuations, local case-ascertainment costs or nationally representative joint parameter data were available. Reference-positive and reference-negative unit costs must include the resources required to obtain eligible validation participants. The joint optimum therefore establishes a conditional design result, not an optimal national recruitment policy. Its prior-dependence and wider-accuracy scenarios examine selected alternatives rather than estimating transportability.

The precision calculations depend on normal and first-order delta approximations, which may be inaccurate near parameter boundaries or with few diagnostic cases. The joint allocation fixes 67 invitations per cluster and assumes outcome-independent response and independent external validation conditional on the design parameters. It excludes informative cluster size, complex weights, threshold-selection uncertainty and correlated validation sampling. Dividing by RR adjusts expected recruitment yield and cannot remove response bias. The cluster simulations generated screening counts directly rather than individual paired screening and reference diagnoses; their known-accuracy analyses were separate from the finite-validation simulations. The simulations evaluate selected departures and do not validate national transportability or every complex survey design. Parameter assurance and prior-predictive width attainment remain distinct, and observed zero or universal event counts do not establish probabilities of exactly zero or one.

## Conclusion

At k = 3, the loss-based rule selected a screening-positive planning proportion of 0.1862 and conditional invitation requirements 39.38% below matched p = 0.5 calculations. Integrating parameter uncertainty increased the screening and current-prevalence invitation targets to 5,346 and 11,866, respectively, with diagnostic accuracy fixed within each current-prevalence scenario. Joint survey–validation allocation increased prior-predictive width attainment from 0.03% to 53.15% at nearly equal assumed cost, and local diagnostic recalibration restored approximately 95% coverage after a specificity shift. Together, these findings show that aligned screening evidence and recruitment losses determine the planning target, while validation allocation and diagnostic calibration shape the precision and validity of current-prevalence estimates.

## Supporting information

S1 Appendix Supplementary methods, proofs, evidence mapping, tables and Fig S1. (PDF)

S2 File Reproducibility code, inputs, numerical outputs and publication figures. (ZIP)
