# Supplementary material for: Decision-theoretic recruitment and validation planning for depression surveys: An illustrative Chinese application
Source: PLoS One. 2026 Oct 1;21(10):e0359867. doi: 10.1371/journal.pone.0359867 (PMC13630222; doi:10.1371/journal.pone.0359867)
Supplement: Supplementary file 1 [file pone.0359867.s001.pdf]

S1 Appendix

Decision-theoretic recruitment and validation planning | PONE-D-26-26922

This appendix specifies the two decision extensions, their mathematical scope, all new sensitivity settings and the finite-validation simulation algorithm. All numerical values below are calculated outputs. The empirical source remains published aggregate information; none of the generated records is participant data.

S1. Bayes recruitment under the complete requirement distribution

Let R be the integer invitation requirement under a coherent joint distribution for all design inputs. Assume R is finite almost surely and $E(R) < \infty$. Let $c_o, c_u > 0$ and consider finite nonnegative integer n with loss $L(n,R) = c_o(n-R)_+ + c_u(R-n)_+$. The expected loss difference between adjacent actions is

$$E\{L(n+1,R) - L(n,R)\} = (c_o + c_u) F_r(n) - c_u .$$

The difference is negative below $q = c_u/(c_o+c_u)$ and nonnegative once $F_r(n) \geq q$. Therefore the smallest integer reaching that CDF level is a global Bayes minimiser. Additional minimisers can occur if the CDF equals q over a gap. This proof does not require independent prior marginals. It applies to the stated loss in invitation units, not to the original continuous loss in the binomial variance term.

The analysis applies this result to the ceiling of Equations (8) and (9) at each original joint-prior draw. Current-prevalence draws use $p_s = Se\pi + (1-Sp)(1-\pi)$, treating diagnostic accuracy as known within a draw. The empirical inverse CDF uses NumPy quantile(method="inverted_cdf") on 1,000,000 integer requirements. Neighbouring integer losses are checked. The theorem is exact; numerical actions approximate the population-prior decision through its empirical distribution.

Table S1. Recruitment-gap Bayes actions under joint parameter uncertainty

k	q	Screening invitations	Current-prevalence invitations
1	0.5000	4,596	10,205
2	0.6667	5,065	11,239
3	0.7500	5,346	11,866
5	0.8333	5,697	12,656
9	0.9000	6,089	13,551
10	0.9091	6,158	13,707
20	0.9524	6,588	14,705

At k=3, empirical mean gap loss falls from 1,600.81 to 1,415.20 for screening and from 5,178.59 to 3,114.42 for current prevalence, compared with the corresponding original conditional candidates. Loss units set $c_o=1$. These comparisons use the same draws and criterion within each estimand.

S2. When the shortfall formulation has no finite risk

For fixed external validation, Equation (11) has no finite solution wherever $V_D \geq d^2J^2/z^2$ and the main-survey numerator is positive. If that event has positive prior probability, defining R=∞ on it gives infinite expected shortfall for every finite n. Omitting those draws or replacing them with an arbitrary large count changes the loss and can manufacture a misleading optimum.

At $\pi_0=28/1{,}045$, Se=Sp=0.86 and validation counts 28/1,017, the first-order floor is 0.0292199. The point lies inside the specified prior support. Continuity yields a neighbourhood with floor above 0.020 and positive prior probability. Thus the fixed-validation shortfall problem cannot be repaired by selecting a higher finite quantile. The joint allocation below uses a different, finite cost-plus-variance criterion and makes that change explicit.

S3. Variance with fixed invitations per cluster

Set b=67 invitations per cluster, approximately the 50/0.75 respondent target rounded upward. Clusters are independent. Conditional on θ, let q_c follow a Beta distribution with mean p and ICC ρ, let $N_c \sim \text{Binomial}(b,RR)$ independently of q_c, and let $Y_c|N_c,q_c \sim \text{Binomial}(N_c,q_c)$. Respondent inclusion is independent of outcomes. Then $E(N_c)=bRR$ and $E[N_c(N_c-1)]=b(b-1)RR^2$. For the ratio estimate $\hat{p}=\Sigma Y_c/\Sigma N_c$,

$$Var(Y_c - pN_c) = p(1-p)\{bRR + \rho b(b-1)RR^2\}.$$

$$Var(\hat{p}) \approx \frac{p(1-p)\{1+(b-1)RR\rho\}}{CbRR}.$$

This is a first-order ratio-estimator variance over C clusters. It accounts for random respondent counts. Replacing its clustering factor with 1+ρ(50−1) under all response values would describe a different design. Here ρ is the ICC of the binary screening outcome.

For independent external reference-positive and reference-negative validation strata, $\hat{Se}$ and $\hat{Sp}$ have conditional binomial variances $Se(1-Se)/n_1$ and $Sp(1-Sp)/n_0$. The Rogan–Gladen gradient is $(1,-\pi,1-\pi)/J$ at a coherent parameter point. Consequently $V(x;\theta)=a_C/C+a_1/n_1+a_0/n_0$, with coefficients given in Equation (14). Prior covariance between parameters does not itself create conditional sampling covariance between independent survey and validation estimators. Prior uncertainty is integrated over the conditional loss; it is not added again as validation-sample variance.

S4. The joint Bayes action and its exact scope

Let $x=(C,n_1,n_0)$, all integers ≥2, and let positive c_j be unit costs. Under the stated first-order loss,

$$\mathscr{R}(x) = E\theta\{\Sigma_j c_j x_j + \lambda V(x;\theta)\} = \Sigma_j[c_j x_j + \frac{\lambda \bar{a}_j}{x_j}], \bar{a}_j = E\theta(a_j).$$

The supports used here bound J away from zero: $J \geq 0.58$ in the base prior and $J \geq 0.35$ in the wider-accuracy scenario. Thus the coefficient expectations are finite. Each term $f_j(x)=c_j x+\lambda\bar{a}_j/x$ has derivative $c_j-\lambda\bar{a}_j/x^2$ and strictly positive second derivative $2\lambda\bar{a}_j/x^3$. Its continuous minimum is $\sqrt{(\lambda\bar{a}_j/c_j)}$. An integer minimum is therefore among the floor and ceiling, clipped at 2. Evaluating those two values separately for each coordinate gives a global minimum over the stated Cartesian integer action space. Ties choose the smaller action.

This proof establishes Bayes optimality for expected first-order variance plus cost. It is not a proof for the exact finite-sample mean squared error of a ratio estimator, for coverage or width-success utility, or for a design allowing arbitrary cluster sizes, weights and correlated validation samples. Future-data simulations assess the chosen action without redefining this loss retrospectively.

For comparison, under a continuous fixed budget B and no active lower bounds, Cauchy–Schwarz gives

$$E(V) \geq \{\Sigma_j \sqrt{(c_j \bar{a}_j)}\}^2 / B.$$

Equality holds at $x_j=B\sqrt{(\bar{a}_j/c_j)}/\Sigma_k\sqrt{(c_k\bar{a}_k)}$. At the optimum the marginal reduction in variance per cost unit, $\bar{a}_j/(c_j x_j^2)$, is equal across components. Figure 4A uses this continuous bound; Table 5 uses integer designs. Integer fixed-budget optimisation is not claimed by that curve.

S5. Priors, cost calibration and sensitivity settings

Table S2. Joint-allocation inputs

Parameter	Base distribution or value	Status
π	Triangular(0.01, 28/1045, 0.10)	Chosen support; published mode
Se	Triangular(0.78, 0.86, 0.90)	Chosen distribution
Sp	Triangular(0.80, 0.86, 0.90)	Chosen distribution
ρ	Triangular(0.02, 0.03, 0.05)	Chosen distribution
RR	Triangular(0.60, 0.75, 0.85)	Chosen distribution
b; cC; c_1; c_0	67; 67; 5; 5	Illustrative design/cost units

Triangular arguments are minimum, mode and maximum. Base marginals are independent. The prior mean of π is approximately 0.04560, which differs from the reference-point mode 0.02679; these are deliberately different evaluations. The prior is an investigator specification, not an empirical posterior. Cost per completed validation participant is all-inclusive and must include case ascertainment. Equal positive and negative unit costs are an illustrative assumption.

Coefficient integration uses exact moments of π, one-dimensional quadrature for response, and two-dimensional Se/Sp quadrature, split at triangular modes. Orders of 32 and 48 nodes per half-support agree to relative tolerance 10^{-11}. The integrated coefficients (aC,a_1,a_0) are (0.0160712500871, 0.000659387009545, 0.235091281432). A separate Monte Carlo estimate is checked against its simulation standard error.

With $v_0=(0.020/1.96)^2$, $\lambda_0=[\Sigma\sqrt{(c_j\bar{a}_j)}/v_0]^2=438{,}057{,}029.134877$. The continuous optimum has $E(V)=v_0$. Integer minimisation yields $1.96\sqrt{E(V)}=0.02000306$, slightly above the target. It would be incorrect to present this penalised optimum as satisfying a hard mean-variance constraint or a 90% probability constraint.

Table S3. Allocation sensitivity to the precision valuation

λ/λ_0	Invitations	n_1	n_0	Relative cost	Parameter assurance
0.25	10,854	120	2,269	22,799	0.0000
0.5	15,343	170	3,209	32,238	0.0363
1	21,708	240	4,538	45,598	0.5372
2	30,686	340	6,418	64,476	0.9759
4	43,416	481	9,077	91,206	1.0000

Additional cost pairs are $(c_1,c_0)=(1,1),(5,5),(20,5),(5,20)$, keeping λ_0 fixed. Prior-dependence scenarios separately impose latent Gaussian-copula correlation −0.5 between Se/Sp or ρ/RR, preserving their triangular marginals. The wider-accuracy scenario uses Se∼Triangular(0.60,0.86,0.95) and Sp∼Triangular(0.75,0.86,0.95). Each prior scenario uses 1,000,000 draws. Full allocations, unchanged-base-design assurance and coefficient Monte Carlo errors appear in joint_design_sensitivity.csv. Very small allocation changes between dependence scenarios should not be interpreted as empirically identified differences.

S6. Future survey and finite-validation simulation

Three designs are evaluated: λ_0; $4\lambda_0$; and a matched-budget comparator retaining validation counts 28/1,017. The comparator buys complete clusters with the remaining budget. It costs 45,559 units, 39 fewer than the base joint allocation. There are five scenarios per design and 10,000 replicates per cell.

Table S4. Finite-validation simulation scenarios

Scenario	Target parameters	Validation parameters
Reference	π=28/1045; Se=Sp=.86; ρ=.03; RR=.75	Seval=Spval=.86
Prior predictive	Draw all five parameters from Table S2	Use that draw's Se,Sp
Specificity shift, source validation	Reference, except Sp=.80	Seval=Spval=.86
Specificity shift, local validation	Reference, except Sp=.80	Seval=.86; Spval=.80
Sensitivity shift, source validation	Reference, except Se=.78	Seval=Spval=.86

For each replicate: (1) obtain θ, calculate p and draw C cluster risks with Beta shapes $p(1/\rho-1)$ and $(1-p)(1/\rho-1)$; (2) draw respondent counts Binomial(67,RR), then positive counts given each respondent count and cluster risk; (3) draw independent validation TP∼Binomial(n_1,Seval) and TN∼Binomial(n_0,Spval); (4) calculate $\hat{p}$, $S\hat{e}=TP/n_1$, $S\hat{p}=TN/n_0$ and $\hat{J}=S\hat{e}+S\hat{p}-1$.

$$\hat{V}s = \frac{C}{C-1}\,\frac{\Sigma_c\left(Y_c - \hat{p}\,N_c\right)^2}{\left(\Sigma_c N_c\right)^2}.$$

The estimate is $\hat{\pi}=(\hat{p}+S\hat{p}-1)/\hat{J}$. The plug-in variance is $[\hat{V}s+\hat{\pi}^2 S\hat{e}(1-S\hat{e})/n_1+(1-\hat{\pi})^2 S\hat{p}(1-S\hat{p})/n_0]/\hat{J}^2$. The interval is $\hat{\pi}\pm1.96\sqrt{\hat{V}}$ and is not truncated to [0,1]. Width attainment means half-width ≤0.020; coverage means the true scenario π lies in that interval. Their intersection, mean bias and MSE are also exported.

Undefined estimates or $\hat{J}\leq0$ count as failures for width and coverage; bias and MSE use valid estimates and explicitly report their valid-sample scope. There were no invalid intervals in the executed cells. The code retains true π, estimated π, half-width and estimated sensitivity/specificity for every replicate, allowing independent reconstruction of all operating characteristics.

New generators use PCG64 with NumPy SeedSequence([20260722,20260907,s]). Stream 1 supplies base allocation draws; streams 2–4 supply the listed prior sensitivities. Stream 19 spawns 15 children, ordered by design (λ_0,$4\lambda_0$,matched budget), then scenario in Table S4 order. Replicate generation is batched in blocks of 128 in the supplied code; reproducing its exact random sequence requires retaining this order. These streams leave all earlier simulations unchanged.

Table S5. Selected finite-validation operating characteristics

Design / scenario	Width	Coverage	Mean bias
λ_0 / prior predictive	0.5315	0.9505	-0.00012
λ_0 / specificity shift unrecalibrated	0.5961	0.0000	+0.08122
λ_0 / specificity shift local validation	0.0004	0.9492	-0.00007
$4\lambda_0$ / prior predictive	1.0000	0.9531	+0.00008
$4\lambda_0$ / specificity shift unrecalibrated	1.0000	0.0000	+0.08110
$4\lambda_0$ / specificity shift local validation	1.0000	0.9483	+0.00011
Fixed validation / prior predictive	0.0003	0.9544	+0.00043
Fixed validation / specificity shift unrecalibrated	0.0000	0.0013	+0.08168
Fixed validation / specificity shift local validation	0.0000	0.9540	-0.00011

A rate based on M=10,000 replicates has Monte Carlo standard error $\sqrt{[\hat{p}(1-\hat{p})/M]}$, at most 0.005. Two-sided 95% Wilson intervals are exported for coverage, width attainment and their intersection. A zero count has upper bound approximately 0.000384; a universal count has lower bound approximately 0.999616. These intervals describe Monte Carlo estimation uncertainty under the stipulated scenario, not transportability.

S7. Accuracy shifts and source-data completeness

Let ΔSe and ΔSp denote target-minus-source changes. At fixed true π, retaining source accuracy in the adjustment gives limiting bias [πΔSe−(1−π)ΔSp]/Jsource. The grid crosses ΔSe∈{0,−0.04,−0.08} with

$\Delta Sp \in \{0, -0.02, -0.06\}$ at π_0. A specificity decrease of 0.06 alone gives bias +0.08110. Larger survey samples reduce variance around that displaced limit; they do not eliminate the displacement.

The rounding enclosure $[0.855, 0.865]^2$ is a numerical source-reporting check only. It is not a confidence region. Conditional on one unweighted complete diagnostic table, the published margins and rounded sensitivity imply TP=24, FN=4, FP=146 and TN=871. The implied PPV is 0.1412 and positive likelihood ratio is approximately 5.9706, inconsistent with reported 0.15 and 5.99 at the stated rounding. These are conditional arithmetic implications, not recovered data, and no inferred exact counts are used to fit a diagnostic posterior.

The accessible source article and PubMed record do not supply participant records sufficient to resolve these differences; the current check did not locate an accessible linked raw dataset. Completing the empirical validation record requires an author-confirmed threshold-specific table, denominators and missingness/weighting details, or authorised de-identified participant data. The reproducibility package is computationally complete for the aggregate-input illustration, while that empirical gap remains.

S8. Field interpretation and file map

Table S6. Practical interpretation of the design analyses

Available evidence / objective	Design implication
Aligned screening data; screening outcome	Use screening positivity directly; specify the planning prior and recruitment-loss ratio.
Current reference prevalence; known target accuracy	Use a coherent π,Se,Sp model and integrate design uncertainty.
Finite independent local validation	Plan C, n_1, n_0 jointly; declare costs and the precision criterion.
Required success probability	Specify it as a design constraint; mean-variance optimality is insufficient.
Accuracy shift or informative response	Calibrate target accuracy and model response; larger recruitment alone does not remove bias.

The original loss-ratio graphic is retained below because it documents sensitivity of the conditional decision. It is supplementary to the joint-allocation comparison in the main text.

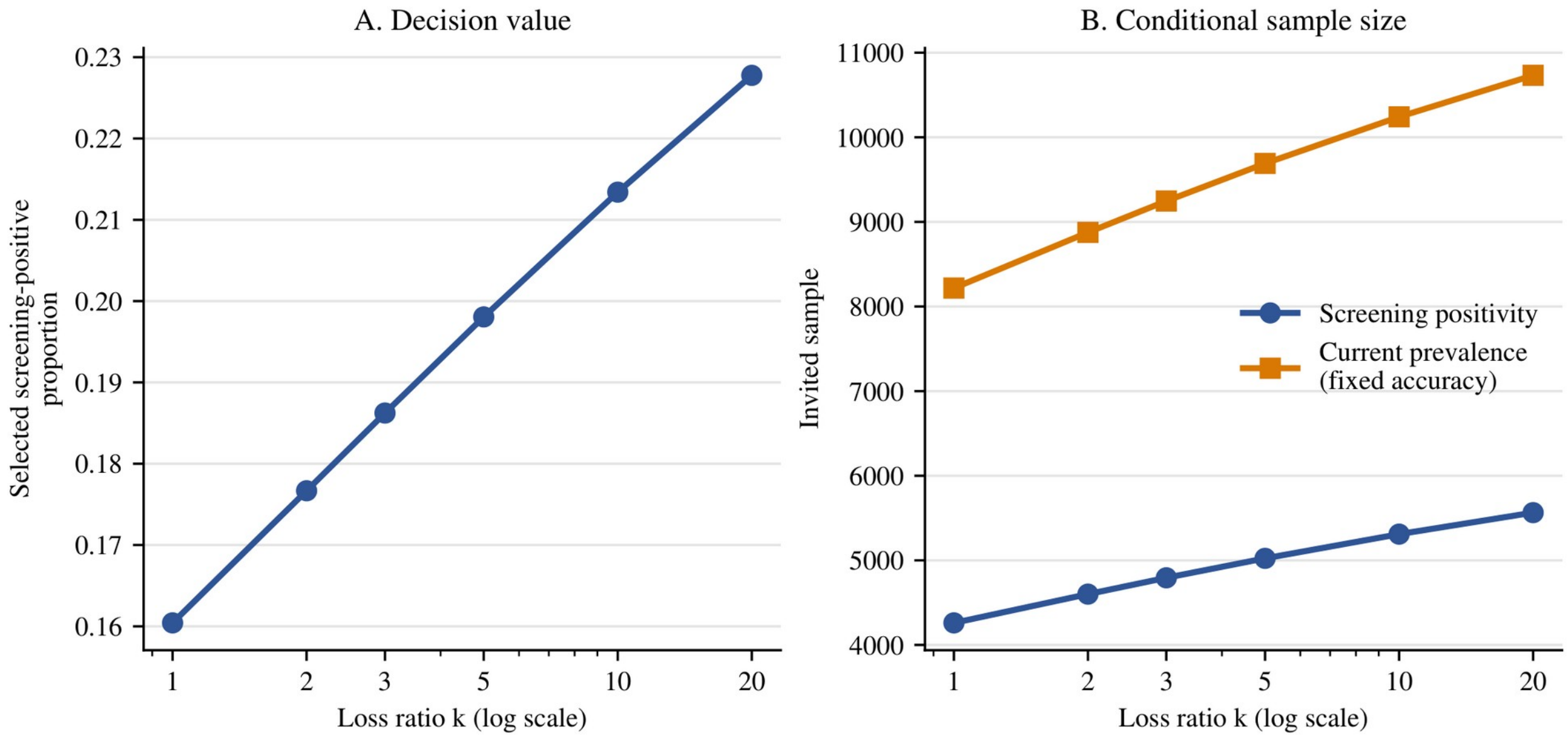

Fig S1. Sensitivity of the planning value and conditional invited sample size to the prospectively specified loss ratio. Panel A shows the selected lower-root screening-positive proportion; Panel B shows the conditional invitation requirements. The six loss ratios are illustrative choices, not data-estimated preferences.

Core files: joint_recruitment_gap_actions.csv (Table S1); table5_joint_designs.csv (allocations); joint_design_sensitivity.csv (cost and prior scenarios); transportability_bias.csv (nine-cell grid); joint_validation_simulations.csv (all 15 future-data cells and Monte Carlo intervals); joint_validation_replicates.npz (replicate-level outputs); results.json (complete parameter and output record). reanalysis_plosone_revised.py regenerates these files and Figures 1–4/S1. Original Tables 1–4 and their diagnostics retain their previous filenames.

S9. Empirical-input and estimand mapping

The numerical analysis separated directly observed screening positivity from current MINI reference classification. Table S7 records the source of each empirical input; the computational input manifest identifies all investigator-specified parameters separately.

Table S7. Source evidence and use in the revised analysis

Input and source	Outcome or source location	Use in the analysis
170 PHQ-9-positive / 1,045 participants [1].	PHQ-9 ≥7; preceding two weeks. Sections 2.3.1 and 3.3.	Direct mean of the screening planning prior: 170/1,045.
28 MINI-positive / 1,045 participants [1].	Current MINI depression reference classification. Sections 2.3.2 and 3.1.	Coherent reference point π_0; mode of the specified triangular π distribution.
Sensitivity 0.86; specificity 0.86 at PHQ-9 ≥7 [1].	Same-study PHQ-9/MINI classification; Table 4. Values rounded to two decimals.	Fixed-accuracy scenarios and modes of the specified accuracy priors; no annual-prevalence mapping.

Input and source	Outcome or source location	Use in the analysis
PHQ-9 followed by MINI assessment [1].	Sections 2.2, 2.3.1 and 2.3.2. Exact elapsed time between assessments is not reported.	Supports defining the reference target as current MINI classification.
12-month MDD prevalence 2.1% (95% CI [1.8%, 2.4%]) [2].	Weighted China Mental Health Survey estimate, adults ≥18 years; Table 2, p. 215.	Introduction background only; absent from all numerical inputs.

The conditional screening prior and the coherent diagnostic reference point serve different calculations. The observed screening mean is 170/1,045 = 0.162679, whereas the rounded-accuracy relation at π_0 gives 0.159292. No adjustment of the source counts was made to force these two values to coincide.

For an annual target, the applicable accuracy parameters would be P(S2w = 1 | D12m = 1) and P(S2w = 0 | D12m = 0). The cited study does not establish these annual-reference parameters. The revised calculation therefore uses the observed screening outcome directly and specifies a separate current-reference target.

S10. Prospective field-elicitation specification

The elicitation template records a small increment in the planned variance term, its additional invitation and field costs, and the consequences of an equal amount of underplanning. Both marginal consequences are valued on one scale to define $k = c_u/c_o$. An alternative specification records an acceptable shortfall probability $1 - q$ and derives $k = q/(1 - q)$. The record includes the setting, participating stakeholders, consequence definitions, central value and sensitivity range before outcome evaluation. This template was provided for subsequent field use; the illustration used investigator-specified k values and did not conduct stakeholder elicitation.

References for Table S7

1. Wang W, Bian Q, Zhao Y, Li X, Wang W, Du J, et al. Reliability and validity of the Chinese version of the Patient Health Questionnaire (PHQ-9) in the general population. Gen Hosp Psychiatry. 2014;36(5):539-544. doi:10.1016/j.genhosppsych.2014.05.021

2. Huang Y, Wang Y, Wang H, Liu Z, Yu X, Yan J, et al. Prevalence of mental disorders in China: A cross-sectional epidemiological study. Lancet Psychiatry. 2019;6(3):211-224. doi:10.1016/s2215-0366(18)30511-x
