# Supplementary material for: Decision-theoretic recruitment and validation planning for depression surveys: An illustrative Chinese application
Source: PLoS One. 2026 Oct 1;21(10):e0359867. doi: 10.1371/journal.pone.0359867 (PMC13630222; doi:10.1371/journal.pone.0359867)
Supplement: Supplementary file 2 [file pone.0359867.s004.docx]

**Response to Reviewers**

**Manuscript ID:** PONE-D-26-26922
**Title:** *A decision-theoretic framework for selecting the planning screening-positive proportion in low-prevalence mental health surveys: application to Chinese depression surveillance*

Dear Academic Editor,

Thank you for the opportunity to revise the manuscript. We have addressed the reviewers’ concerns about the estimand, interpretation of the calculated sample-size difference, evidential basis of the Beta prior and loss ratio, transportability of PHQ-9 diagnostic accuracy, and assumptions underlying the sample-size equations. These changes required reanalysis as well as revision of the text.

The primary research question remains unchanged: the manuscript evaluates how prior evidence and asymmetric losses can be used to select a planning screening-positive proportion, $p_{s}^{*}$, and quantifies the corresponding invited sample size relative to the conventional $p=0.5$ calculation in a prespecified Chinese depression-surveillance application. The loss ratio $k=c_{u}/c_{o}$ is now treated explicitly as an externally specified policy input that should be elicited before sample-size results are examined. Because the present application contains no such elicitation data, $k=3$ is retained solely as an illustrative base case and is not described as an empirically optimal value.

The revision addresses each substantive point raised by the editor and reviewers. Reference-standard disorder prevalence $\pi$ is distinguished throughout from the PHQ-9 screening-positive proportion $p_{s}$, with separate sample-size equations for the two estimands. The 42.85% difference is reported only as a fixed-input illustration conditional on $k=3$ and fixed diagnostic accuracy. Beta(15,85) is treated as an evidence-informed planning distribution with prespecified effective sample size, not as a prior estimated from three independent sources. Uncertainty in sensitivity and specificity is propagated through the delta method, including covariance terms for dependent validation designs. The base value $k=3$ is identified as illustrative, while six prespecified $k$ values are compared without designating a post hoc “best” value. A prospective loss-elicitation protocol has been added. The assumptions governing clustering, unequal cluster size, non-response, diagnostic transportability, parameter dependence and external or nested validation are also stated and examined explicitly.

All numerical results were recomputed from the accompanying reproducibility scripts. Use of the exact Beta transformation and rounding only after the complete calculation changed the base invited sample from 8,718 to 8,717; the conditional percentage difference remains 42.85% after rounding to two decimal places.

# Academic Editor

## Comment

*The Conclusions should bring the principal findings and their implications together in one place, even where individual points are also discussed elsewhere.*

### Response

The Conclusions now comprise three inferentially distinct statements rather than a second presentation of the Results. The first answers the research question by reporting the selected $p_{s}^{*}$ and the estimand-specific implication for recruitment. The second retains the principal counter-result: the conditional differences from $p=0.5$ did not constitute robust efficiency gains because no candidate $k=1$–20 attained the prespecified 90% joint-assurance criterion, and the corrected-prevalence precision target was unattainable by enlarging only the main survey once published validation uncertainty was propagated. The third advances one directly supported planning implication: $k$, local prior updating and estimand-specific assurance should be resolved before recruitment is fixed. General limitations concerning prior construction, transportability, clustering and informative response remain confined to the dedicated Limitations subsection.

The Abstract has been aligned with the same evidential sequence. Its Results identify the model associated with each numerical finding, whereas its Conclusions state the supported answer, the principal counter-result and the corresponding prospective planning rule.

# Reviewer 1

## Comment 1

*The background section should more clearly distinguish between true disorder prevalence and the screening-positive rate.*

### Response

This distinction has been made explicit from the Introduction onward and is maintained consistently in the Methods, Results, Discussion, tables and figure captions. The revised manuscript defines

$$\pi=\text{reference-standard MDD prevalence}$$

and

$$p_{s}=P\left( PHQ - 9\geq7 \right)=\mathrm{Se} \pi+\left( 1-\mathrm{Sp} \right)\left( 1-\pi\right).$$

The corresponding sample-size problems are now separated. If the estimand is the PHQ-9 screening-positive proportion $p_{s}$, no Rogan-Gladen correction or diagnostic inflation factor is applied. If the estimand is corrected MDD prevalence $\pi$, the sampling variance is transformed through $J^{-2}=\left( Se+Sp-1 \right)^{-2}$, and uncertainty in $Se$ and $Sp$ is propagated.

### Changes made

- A definitional paragraph and Equation (1) were added to the Introduction.
- Equations (8) and (9) now give separate invited-sample calculations for the PHQ-9 screening-positive proportion, $n_{inv,s}$, and corrected MDD prevalence when sensitivity and specificity are treated as fixed, $n_{inv,\pi}^{\left( fixed \right)}$.
- Table 1 reports sample sizes separately for the screening-positive and corrected-prevalence estimands.
- Terminology was standardised to “reference-standard disorder prevalence,” “screening-positive proportion,” “planning screening-positive proportion,” and “corrected prevalence.”

## Comment 2

*The research question should be presented earlier and more explicitly.*

### Response

The research question is now stated directly at the end of the Introduction, before the Methods. It preserves the original focus on selecting $p_{s}^{*}$ and comparing the resulting sample size with $p=0.5$. Three evaluation questions are then specified: the base-case planning value and invited sample size; the conditional sample-size difference from $p=0.5$; and sensitivity to the prior, diagnostic accuracy, clustering, response and loss assumptions.

The manuscript also clarifies that the loss rule answers the original question by generating $p_{s}^{*}$, whereas assurance, coverage and failure-boundary analyses evaluate the robustness of the resulting conditional design. They do not constitute alternative criteria for estimating or selecting $k$. This preserves the original research question while preventing a conditionally calculated sample from being presented as universally adequate.

### Changes made

- The final paragraph of the Introduction now states the primary research question and the three evaluation questions explicitly.
- The Abstract and Conclusions follow the same order: the selected $p_{s}^{*}$ and estimand-specific invitation requirement, the robustness of the conditional comparison with $p=0.5$, and the prospective planning implication.

## Comment 3

*The Discussion should further explore the practical value of the proposed methods, including applicable contexts and implications for practice.*

### Response

The revised Discussion now translates the statistical results into a staged planning procedure. The practical recommendations begin with explicit selection of the estimand, then distinguish settings with precise local diagnostic validation from those relying on external $Se/Sp$, and finally address pilot updating, uncertain ICC, unequal cluster sizes and informative non-response. The manuscript also identifies conditions under which increasing the main-survey sample is ineffective because diagnostic validation or selection bias is the binding source of error.

### Changes made

- A dedicated practical-application paragraph was added to the Discussion.
- Table 5 provides decision rules for six common field situations, including screening-positive surveys, corrected-prevalence surveys, external validation, low specificity, poorly characterised clustering/response and informative non-response.
- The Methods provide a formal local-pilot update and guidance for prior-data conflict.
- Analytical boundaries identify the maximum screening-positive proportion, maximum ICC and minimum response rate compatible with the base 8,717-person design.

## Comment 4

*Some parts of the Discussion are repetitive. The limitations are comprehensive but list-like and should be organised more logically.*

### Response

The Discussion has been reorganised as a cumulative argument. The opening paragraph answers the research question and states the estimand-specific $k=3$ calculations. The next paragraphs define the integrative contribution, interpret the diagnostic-validation precision floor, explain why $k$ requires prospective elicitation, and give implementation rules. A dedicated Limitations subsection then organises limitations by prior, measurement and survey-model misspecification. The Conclusions distinguish the supported base-case answer from the principal robustness counter-result and then state one directly corresponding planning implication.

Repeated statements that the 42.85% result is “conditional,” “not universal” or requires “local recalibration” have been consolidated into the opening and closing interpretive statements. This produces a shorter and more logically cumulative Discussion.

### Changes made

- The previous seven-item limitations list was replaced by three analytically coherent categories.
- Repetitive policy and transferability statements were removed.
- Each Discussion paragraph now begins with a substantive conclusion and develops one principal claim.
- The Conclusions now provide a concise three-sentence synthesis of the supported answer, the principal counter-result and its direct planning implication; general limitations are confined to the dedicated subsection.

## Comment 5

*The author should carefully review and revise the formatting of the entire manuscript, including line spacing and text alignment, in accordance with the journal’s formatting guidelines.*

### Response

The manuscript has been reformatted throughout. The revised files use a single-column layout, double-spaced main text, left alignment, one-inch margins, continuous line numbering, page numbering and no more than three heading levels. Equations are supplied as editable Word equations rather than plain-text approximations. Tables use consistent geometry and follow their first textual citation; figure captions and figures likewise follow their first citation. APA author-date citations and the APA-formatted reference list have been standardised consistently, as requested for this revision.

### Changes made

- The entire manuscript was re-typeset in a consistent academic format.
- Mathematical notation, table titles, table notes, figure captions and abbreviations were standardised.
- A marked-up copy and an unmarked copy are supplied as separate files.

# Reviewer 2

## Comment 1

*The reported 42.85% sample-size reduction is overstated. Simulations show that* $p^{*}=0.173$ *fails the precision target under higher prevalence, lower specificity or adverse parameters, while* $p=0.5$ *often succeeds; the gain is highly conditional.*

### Response

The interpretation has been revised accordingly. The manuscript no longer presents 42.85% as a general reduction. It is now defined as a base-case calculated difference between two planning proportions within the same fixed-input formula. At illustrative $k=3$, the samples are 4,519 for screening positivity and 8,717 for corrected prevalence when diagnostic accuracy is treated as fixed, compared with 7,908 and 15,254 under $p=0.5$. Across $k=1,2,3,5,10$ and 20, $p_{s}^{*}$ ranged from 0.1477 to 0.2132 and the conditional differences narrowed from approximately 49.7% to 32.9%. The manuscript therefore reports a transparent mapping from prespecified loss preference to sample size rather than replacing the original claim with another universal percentage.

The robustness analysis was also strengthened. Screening outcomes are generated at cluster level through a beta-binomial model, and standard errors are estimated from cluster residual totals rather than from the planning approximation. The design-effect parameter $\bar{m}=50$ is now defined explicitly as the anticipated number of analysed respondents per cluster; invitations are allocated so that the planning response rate yields this respondent cluster size. After alignment of the analytical and simulation definitions, the 8,717-person design attained the half-width target in 75.78% of base-case replicates, but in only 12.97% at $\pi=0.10$, 0.71% at $Sp=0.80$, 17.24% at $Se=0.78$, and 1.98% at $\rho=0.05$ with $RR=0.60$. The corresponding probabilities for the $p=0.5$ design were 98.96%–100% in these single-departure scenarios. Under the combined adverse condition, the $p=0.5$ design attained the target in 3.20% of replicates.

### Changes made

- “Sample-size reduction” was replaced where necessary by “base-case calculated difference” or “conditional difference.”
- The 42.85% value is no longer the sole headline conclusion.
- Equation (12) defines joint assurance as a robustness measure for a sample generated by a prespecified $k$; it is not used to select $k$.
- Table 2 compares the planning value, invited sample size and joint assurance of every candidate $k$; Figure 4 displays the loss-ratio sensitivity of $p_{s}^{*}$ and both estimand-specific sample sizes.
- Table 4 and Figure 3 report scenario-specific precision assurance and coverage.
- Exact one-parameter validity boundaries are reported for the 8,717-person design.

## Comment 2

*The Beta(15,85) prior relies on only three sources and shifts substantially with moderate heterogeneity, yet guidance for updating is limited.*

### Response

The evidential status of the prior has been corrected. The CMHS is used as the nationally representative location anchor. Gu et al. (2013) and Zhao et al. (2021) are used for triangulation and range assessment rather than described as independent evidence units. The revised manuscript explicitly notes possible overlap between the meta-analyses.

Beta(15,85) is now defined as a planning prior with mean 0.15 and prespecified ESS 100. The ESS is not claimed to be estimated from the three sources. Sensitivity analyses vary ESS from 5 to 200, omit each source in turn, and vary predictive logit-scale heterogeneity from 0.05 to 0.75. At $\tau=0.30$, $p_{s}^{*}$ increased to 0.1826 and the invited sample to 9,106; at $\tau=0.75$, the corresponding values were 0.2325 and 10,888.

Updating guidance has been made operational. For an approximately independent local pilot with $x$ positives among $n$ participants, the prior updates to $\mathrm{Beta}\left( \alpha+x,\beta+n-x \right)$. For a clustered or weighted pilot, both binomial counts are adjusted: $x_{eff}=n_{eff}\hat{p}_{s,w}$, giving $\mathrm{Beta}\left( \alpha+x_{eff},\beta+n_{eff}-x_{eff} \right)$. A design-consistent hierarchical analysis is recommended when a single ESS is inadequate. The manuscript also instructs planners to examine prior-predictive conflict and, when conflict is material, discount the ESS or use a robust-mixture prior.

### Changes made

- The phrases “empirically calibrated from three independent sources” and “empirical-Bayes prior” were removed.
- The revised Methods distinguish prior location, prior strength and predictive heterogeneity.
- ESS, leave-one-source-out and heterogeneity sensitivities are reported in the Results and Figure 1A.
- Equation (6) and accompanying text provide local updating and conflict-management guidance.

## Comment 3

*The framework depends critically on transportability of* $Se=Sp=0.86$*. When specificity falls to 0.80, precision fails and coverage collapses if original values are assumed; this requires stronger emphasis.*

### Response

Diagnostic transportability is now a principal result rather than a brief limitation. Specificity recalibration updates both the expected $p_{s}$ prior and the Youden-index variance term. Reducing $Sp$ from 0.86 to 0.80 increased the CMHS-anchored prior mean from 0.1551 to 0.2139 and the fixed-accuracy invited sample from 8,934 to 13,260.

More importantly, the revised analysis propagates sampling uncertainty from the validation study. Wang et al. (2014) included 1,045 participants but only 28 reference-standard MDD cases. Under independent binomial approximation, the diagnostic-accuracy component alone produces a minimum attainable 95% CI half-width of approximately 0.0292 for $\pi=0.021$. This exceeds the prespecified 0.020 target; no finite increase in the main-survey sample can satisfy the target unless the diagnostic validation information is strengthened or modelled differently.

Simulation additionally confirms that when true specificity is 0.80 but the analysis retains $Se=Sp=0.86$, coverage is zero for all three sample-size strategies. This is identified explicitly as bias caused by diagnostic misspecification rather than inadequate recruitment.

### Changes made

- Equation (10) gives the first-order delta-method variance, including the covariance terms needed for dependent validation designs.
- Equation (11) gives the invited main-survey sample size for the independent external-validation case and states its feasibility condition. Its notation was changed from $n_{\pi,full}$ to $n_{inv,\pi}^{\left( ext \right)}$ to avoid implying that the equation covers nested validation or every source of uncertainty.
- Table 3 and Figure 1B report specificity recalibration.
- Figure 2 reports the diagnostic-validation precision floor.
- The Abstract Results, main Results, Discussion and dedicated Limitations subsection now state that additional main-survey recruitment cannot correct non-transportable diagnostic accuracy; this limitation is not repeated in the Conclusions.

## Comment 4

*The cost ratio* $k=3$ *is arbitrary, lacks an elicitation protocol, and materially affects results.*

### Response

The revised manuscript agrees that $k=3$ has no direct empirical or literature-based justification. It is retained only as an illustrative base-case policy weight. References to prevalence-survey guidance as support for the numerical value 3 have been removed. The decision-theory references support the quantile relationship $q=k/\left( 1+k \right)$, not the choice of $k=3$.

Sensitivity analysis now spans $k=1,2,3,5,10$ and 20. Across this range, $p_{s}^{*}$ varied from 0.1477 to 0.2132; screening-positive samples ranged from 3,981 to 5,306 and fixed-accuracy corrected-prevalence samples from 7,680 to 10,236. The conditional difference from $p=0.5$ narrowed from approximately 49.7% to 32.9%. Under the primary joint planning distribution, assurance increased from 37.80% to 81.91% for screening positivity and from 10.55% to 51.68% for corrected prevalence. The manuscript does not designate an empirically “best” $k$, because neither prevalence data nor the resulting sample size identifies the relative policy loss.

The Methods now provide a prospective elicitation protocol. Investigators first define a common increment in the information term and quantify the marginal consequences of over- and underplanning on a common monetary or utility scale. Survey commissioners, field investigators and end users then determine $k=c_{u}/c_{o}$, document the consequence definitions and stakeholder roles, and prespecify a plausible low-to-high range before viewing the resulting sample sizes. If commensurate valuation is not credible, stakeholders may instead specify the largest acceptable prior probability that the required information term exceeds the planned value and derive $k$ from that probability. In the present application, no elicitation data exist; accordingly, $k=3$ remains explicitly illustrative rather than being presented as the output of this protocol.

### Changes made

- $k=3$ is labelled “illustrative” in the Abstract, Methods, Results, Discussion and Conclusions.
- Unsupported literature justification for the numerical value was removed.
- Table 2 reports candidate $p_{s}^{*}$, invited sample size and joint assurance for every $k$.
- No $k$ is selected post hoc from the most attractive sample-size or assurance result.
- A prospective field elicitation protocol, including documentation and sensitivity-analysis requirements, was added to the Methods.

## Comment 5

*Equation (8) applies unquantified independence assumptions. The Abstract should qualify results as base-case conditional, the contribution should be framed as integrative, and full code deposition with minor figure/terminology improvements are needed.*

### Response 5a: assumptions underlying Equation (8)

The earlier independence statement was imprecise and has been removed. Multiplying fixed scalar values of $DEFF$, $J^{-2}$ and ${RR}^{-1}$ does not require probabilistic independence. The actual assumptions are now stated: the design effect must capture clustering, weighting and unequal cluster sizes; non-response must be ignorable after the planned weighting; diagnostic accuracy must transport across regions, clusters and response groups; validation and survey estimators must be independent when covariance terms are omitted; and the final analysis must use the diagnostic model assumed in planning.

The joint-uncertainty analysis makes its distributional assumptions explicit. The primary calculation uses independent triangular planning distributions because joint parameter data are unavailable. Uniform-marginal and adverse-rank analyses evaluate distributional form and dependence, while the cluster simulations separately examine higher prevalence, lower specificity, lower sensitivity, adverse ICC/response and their combination. These analyses quantify robustness; they do not estimate $k$ or convert an illustrative calculation into an unconditional recommendation.

The design effect allows unequal cluster sizes through

$$\mathrm{DEFF}=1+\rho\left[ \left( 1+\mathrm{CV}_{m}^{2} \right)\bar{m}-1 \right].$$

The delta-method derivation now separates survey–validation covariance from covariance between $\hat{Se}$ and $\hat{Sp}$. Independence of an external validation study from the main survey sets $\mathrm{Cov}\left( \hat{p}_{s},\hat{Se} \right)$ and $\mathrm{Cov}\left( \hat{p}_{s},\hat{Sp} \right)$ to zero, but does not by itself imply $\mathrm{Cov}\left( \hat{Se},\hat{Sp} \right)=0$. The latter is set to zero only under the stated independent-binomial validation approximation. Equation (11) now states the additional regularity condition $J>0$, its finite-solution condition, and its restriction to external validation whose variance is fixed with respect to the main-survey sample. The informative-response simulation further shows that nominal precision may coexist with substantial bias: the half-width criterion was attained in 99.43% of replicates for the $p_{s}^{*}$ design, whereas coverage was 55.30%.

**Location in revised manuscript:** Methods, “Estimand-specific sample-size formulae,” “Diagnostic-accuracy uncertainty and the precision floor,” “Assumptions underlying the composite design calculation,” “Joint-uncertainty robustness analysis,” and “Cluster simulation operating characteristics.”

### Response 5b: qualification of the Abstract

The Abstract now labels $k=3$ as illustrative, reports the 42.86% and 42.85% differences only under the prespecified base-case formulae, and distinguishes the 4,519-person screening-positive design from the 8,717-person fixed-accuracy calculation for corrected prevalence. The Results identify asymmetric-loss selection, base-case calculation, loss-ratio sensitivity, joint-uncertainty assurance and propagated validation uncertainty as separate analyses. The Conclusions then state that the numerical differences from $p=0.5$ were not robust efficiency gains, because no candidate $k$ attained 90% joint assurance and the corrected-prevalence precision target was not recoverable by increasing only the main survey.

**Location in revised manuscript:** Abstract, Methods, Results, Discussion, Limitations, and Conclusions.

### Response 5c: framing of the methodological contribution

The revision does not claim a new Bayesian quantile rule or a new decision-theoretic theorem. The Introduction and Discussion identify the contribution as the integration of established asymmetric-loss selection with prospective loss specification, estimand-specific sample-size calculations, diagnostic-validation uncertainty, clustering, response adjustment and robustness analysis.

**Location in revised manuscript:** final two paragraphs of the Introduction and second paragraph of the Discussion.

### Response 5d: deposition and reproducibility of the code

The accompanying reproducibility package contains the complete Python analysis, fixed random seed, input parameters, machine-readable outputs and nine unit checks. The tests verify the base decision value, both estimand-specific sample sizes, the delta-method gradient, the validation precision floor, the external-validation feasibility calculation, joint assurance, and consistency between the respondent cluster-size definition in Equation (7) and the simulated invitation allocation.

**Location in revised manuscript:** Methods, final paragraph; Data and code availability; accompanying reproducibility package.

### Response 5e: figures and terminology

The title, main text, tables and figure captions now distinguish “screening-positive proportion,” “reference-standard prevalence,” “planning value,” “conditional difference” and “assurance.” Figure 1 presents prior-strength and specificity-recalibration analyses; Figure 2 presents the diagnostic-validation precision floor; Figure 3 presents scenario-specific precision assurance and coverage; and Figure 4 presents loss-ratio sensitivity. The figures use consistent panel labelling, non-italic bold figure labels in the manuscript, and estimand-specific axis terminology.

**Location in revised manuscript:** title and terminology throughout; Figures 1–4 and their captions.

We appreciate the reviewers’ careful evaluation. Their comments materially improved the manuscript by aligning its estimand, equations, evidence claims and conclusions and by converting the principal numerical result from an overgeneralised efficiency statement into a fully specified and testable design comparison.

Sincerely,

Han Liu
