# Supplementary material for: Decision-theoretic recruitment and validation planning for depression surveys: An illustrative Chinese application
Source: PLoS One. 2026 Oct 1;21(10):e0359867. doi: 10.1371/journal.pone.0359867 (PMC13630222; doi:10.1371/journal.pone.0359867)
Supplement: Supplementary file 3 [file pone.0359867.s005.docx]

Response to Reviewers

Manuscript PONE-D-26-26922 | Revised 8 September 2026

Dear Dr Lin,

Thank you for the further review. The revision replaces the annual-prevalence-based screening anchor and updates the dependent calculations. The principal changes and the response to the remaining temporal-alignment concern are detailed below.

Page and line references identify the PLOS submission edition (Manuscript.docx; 28-page clean rendering) dated 8 September 2026. Supplementary page references identify S1 Appendix. The marked manuscript records changes relative to the uploaded R1 clean manuscript.

# Summary of the main revisions

**Evidence and outcomes.** The screening prior now uses observed PHQ-9 positivity. The 12-month national estimate is retained solely as background, and current MINI-defined prevalence is analysed as a separate outcome. See Methods, pp. 4–6, and S1 Appendix, Section S9, pp. 7–8.

**Recalculation and diagnostic validation.** Revised inputs were propagated through the sample-size calculations, sensitivity analyses and simulations. Joint recruitment–validation allocation and its operating characteristics are reported separately from conditional planning. See Results, pp. 14–21, Tables 1–5 and Figures 1–4.

**Reporting and interpretation.** Methods specifies the executed analysis steps; Results follows the calculation sequence; Discussion interprets those results; and the Conclusion answers the study aim. See Methods, pp. 4–14; Discussion, pp. 21–23; and Conclusion, p. 24.

**References and reproducibility.** References and source-to-input links were checked. The package includes the original-R1 Word comparison, a clean manuscript, the supporting derivations, and executable code with numerical and simulation outputs.

# Reviewer 1: the 12-month prevalence and two-week PHQ-9 concern

**Comment.**

The author has responded carefully to the comments raised in the previous round of review, and the revised manuscript has improved substantially. However, one issue still requires clarification. The manuscript uses the 12-month MDD prevalence estimate, together with the sensitivity and specificity of the PHQ-9, to anchor the planning distribution for the expected PHQ-9 screening-positive proportion. The standard PHQ-9, however, primarily assesses depressive symptoms experienced over the past two weeks. It is therefore unclear whether the PHQ-9 screening-positive proportion and the 12-month reference-standard MDD prevalence are temporally aligned estimands and whether the cited sensitivity and specificity estimates support this mapping.

**Response.** The original mapping lacked accuracy estimates validated against a 12-month reference target. The correction therefore changes the empirical anchor, outcome definitions and dependent analyses. It is implemented in Methods and Results, with the remaining source limitations identified separately.

## 1. The annual prevalence estimate was removed from the analysis inputs

The national 12-month MDD estimate no longer enters prior construction, specificity recalibration, parameter draws or simulation scenarios. The Introduction retains the weighted estimate of 2.1% as epidemiological background and corrects its 95% confidence interval to 1.8%–2.4%, as reported in Table 2 of Huang et al. (2019). The prior construction no longer triangulates screening yield from annual prevalence sources.

**Location.** Introduction, p. 3, lines 37–43; Methods, “Planning prior and updating,” pp. 5–6, lines 107–110; S1 Appendix, Section S9 and Table S7, pp. 7–8.

## 2. The screening prior was rebuilt from directly observed PHQ-9 results

At PHQ-9 ≥7, the Shanghai validation study reported 170 screening-positive results among 1,045 community participants (Wang et al., 2014, Section 3.3 and Table 1). The revised screening prior uses their observed proportion, 170/1,045 = 0.162679, without converting a disorder-prevalence estimate into screening positivity. Retaining the specified ESS of 100 gives Beta(16.2679426, 83.7320574), replacing Beta(15, 85). ESS remains an investigator-specified discount rather than an estimated national sample size.

**Location.** Methods, “Planning prior and updating,” pp. 5–6, lines 107–110; Figure 1A; S1 Appendix, Table S7, pp. 7–8; input_provenance.csv.

## 3. The reference-prevalence analysis was defined separately and indexed to its target

Methods now distinguishes S2w, PHQ-9 positivity for symptoms over the preceding two weeks, from Dcur, the current MINI depression reference classification. Equation (1) uses Se = P(S2w = 1 | Dcur = 1) and Sp = P(S2w = 0 | Dcur = 0). The same study reported 28 MINI-positive participants and threshold-specific sensitivity and specificity of 0.86 (Wang et al., 2014, Sections 3.1 and 3.3; Table 4). These aggregates define the current-reference illustration; current-prevalence parameter draws generate screening positivity from Equation (1).

The source reports PHQ-9 administration followed by MINI assessment and describes the MINI reference in relation to current major depressive episodes (Wang et al., 2014, Sections 2.2–2.3.2). Its two-week follow-up in 100 participants concerned PHQ-9 test–retest reliability (Wang et al., 2014, Section 2.2). That follow-up does not establish the elapsed interval between PHQ-9 and MINI administration, which the source does not specify.

A 12-month prevalence analysis would instead require sensitivity P(S2w = 1 | D12m = 1) and specificity P(S2w = 0 | D12m = 0), where D12m identifies a reference-standard episode during that year. Such annual-reference accuracy was not established by the cited study. The revised numerical analysis therefore concerns directly observed screening positivity and a separate current reference outcome; neither is presented as 12-month MDD prevalence.

**Location.** Methods, “Estimands and measurement relation,” p. 4, lines 73–84; Equation (1); Methods, “Joint parameter assurance,” p. 10, lines 198–214; S1 Appendix, Section S9, pp. 7–8; Limitations, p. 23, lines 479–486.

## 4. All dependent numerical results were updated

At the unchanged illustrative loss ratio k = 3, the revised planning proportion is 0.1862346, compared with 0.1727 in R1. Conditional screening invitations increased from 4,519 to 4,794, and current-reference invitations with fixed diagnostic accuracy increased from 8,717 to 9,247. The updated differences from matched p = 0.5 calculations are 39.38%. Integrating uncertainty over the full invitation requirement separately selects 5,346 screening and 11,866 current-reference invitations, with diagnostic accuracy fixed within each current-reference scenario. Sensitivity analyses, simulations, figures, the Abstract and the Conclusion use the revised inputs and outputs.

**Location.** Results, “Planning value and base-case sample sizes,” p. 14, lines 300–309; Table 1; Results, pp. 15–21; Abstract, p. 2; Conclusion, p. 24, lines 505–513; S1 Appendix, Table S1.

## 5. The empirical evidence and unresolved source details were made traceable

Table S7 maps each count, diagnostic-accuracy estimate and time-window definition to its source section and analytical use. The directly observed screening mean, 0.162679, is distinguished from the model-implied reference-point probability, 0.159292, calculated using 28/1,045 and rounded Se = Sp = 0.86. Source counts were not altered to force agreement. The source audit also records inconsistencies among published diagnostic summaries. No author-confirmed complete diagnostic table or participant-level dataset is claimed. Sampling uncertainty and population transportability are evaluated separately from numerical rounding.

**Location.** S1 Appendix, Section S7, p. 5, and Section S9/Table S7, pp. 7–8; Limitations, p. 23, lines 479–486; input_provenance.csv.

# Reviewer 2

**Comment.** “The revised version is acceptable and the necessary corrections have been made.”

**Response.** Thank you for this assessment. The additional changes prompted by Reviewer 1 have been checked for consistency across the manuscript, supporting analyses and figures.

# Further revisions and journal requirements

## Methods, decision criteria and validation precision

The study aim is stated at the end of the Introduction. Methods specifies the input distributions, loss ratios, sensitivity grids, random streams, estimators and performance metrics. Conditional recruitment, parameter assurance and simulated interval-width attainment are distinguished. Equations (10)–(11) define the independent-validation variance budget and its feasibility boundary; the reference-point half-width floor is 0.0292, above the 0.020 target. Equations (13)–(15) add decisions over the full recruitment requirement and joint survey–validation allocation, with proofs for the stated objectives in S1 Appendix.

Joint allocation achieved 53.15% prior-predictive width attainment, compared with 0.03% when a nearly equal assumed budget retained the original validation counts. Costs, priors and precision valuations are identified as assumptions. The Discussion distinguishes optimality for the specified risk from attaining a prespecified probability of adequate precision. Operational steps are reported in Methods; response bias, simulation scope and transportability restrictions appear in Limitations.

**Location.** Introduction, p. 4, lines 66–70; Methods, pp. 7–14; Methods, “Bayes decisions under joint uncertainty” and “Joint allocation of survey and validation samples,” pp. 10–12, lines 215–251; Equations (13)–(15); Table 5 and Figure 4; Discussion, pp. 21–23, lines 436–477; Limitations, pp. 23–24, lines 479–503.

## Results, figures, Discussion and Conclusion

**Reporting request.** “The results section is difficult to follow in its current form. Key findings should be presented more clearly and systematically, with improved figure quality and more transparent reporting.”

Results now proceeds from the planning value and conditional invitation counts to joint parameter assurance, prior sensitivity, diagnostic-validation precision, simulation performance and joint allocation. Tables identify the estimand and precision metric, and the long simulation passages are divided by function. Discussion interprets the corresponding findings. The Conclusion answers the research aim using the recalculated recruitment, validation-allocation and calibration results. Figures 1–4 and S1 are supplied as 600 dpi TIFFs with vector PDF copies; simulation figures include Monte Carlo intervals. Table labels are non-italic, and the full titles of Figures 1 and 2 are bold.

**Location.** Results, pp. 14–21, Tables 1–5 and Figures 1–4; Discussion, pp. 21–23, lines 436–477; Conclusion, p. 24, lines 505–513.

## Reference corrections

All 21 manuscript references were matched to the in-text citations in both directions. Grouped literature citations were replaced by one source per cited sentence. The revised bibliography adds the original PHQ-9 instrument paper, a two-phase prevalence-estimation paper and a study of selective cutoff reporting. Two prevalence reviews used in the former annual-prevalence triangulation and one unused general decision-theory source were removed. Source scope and bibliographic details were checked, including the national prevalence confidence interval and the Shanghai assessment protocol. The PLOS manuscript uses numbered references in order of first appearance; the APA edition uses author–year citations.

**Location.** Introduction and References; citation_statement_map.csv; citation_audit.csv; S1 Appendix, Table S7.

## Submission files and reproducibility

The package supplies separate files labelled Response to Reviewers, Revised Manuscript with Track Changes and Manuscript. The native Word comparison uses the original clean R1 manuscript corresponding to pages 8–33 of the uploaded submission PDF. Accepting its content revisions reproduces the current clean manuscript; rejecting them restores the R1 content. The comparison is reconstructed from the two versions and retains the revised page layout.

S1 Appendix supplies the derivations, simulation specifications and source mapping. S2_Reproducibility.zip contains the executed analysis script, software requirements, numerical outputs and replicate arrays. The computational record reproduces the declared aggregate-input illustration and distinguishes simulated observations from participant data. The funding and competing-interest declarations are retained.

**Location.** Data and code availability, p. 25, lines 521–526; Declarations; S1 Appendix; S2_Reproducibility.zip; original_comparison.json.

Sincerely,
Han Liu
