# Supplementary material for: Decision-theoretic recruitment and validation planning for depression surveys: An illustrative Chinese application
Source: PLoS One. 2026 Oct 1;21(10):e0359867. doi: 10.1371/journal.pone.0359867 (PMC13630222; doi:10.1371/journal.pone.0359867)
Supplement: Supplementary file 4 [file pone.0359867.s002.zip › S2_Reproducibility/results/figure1.pdf]

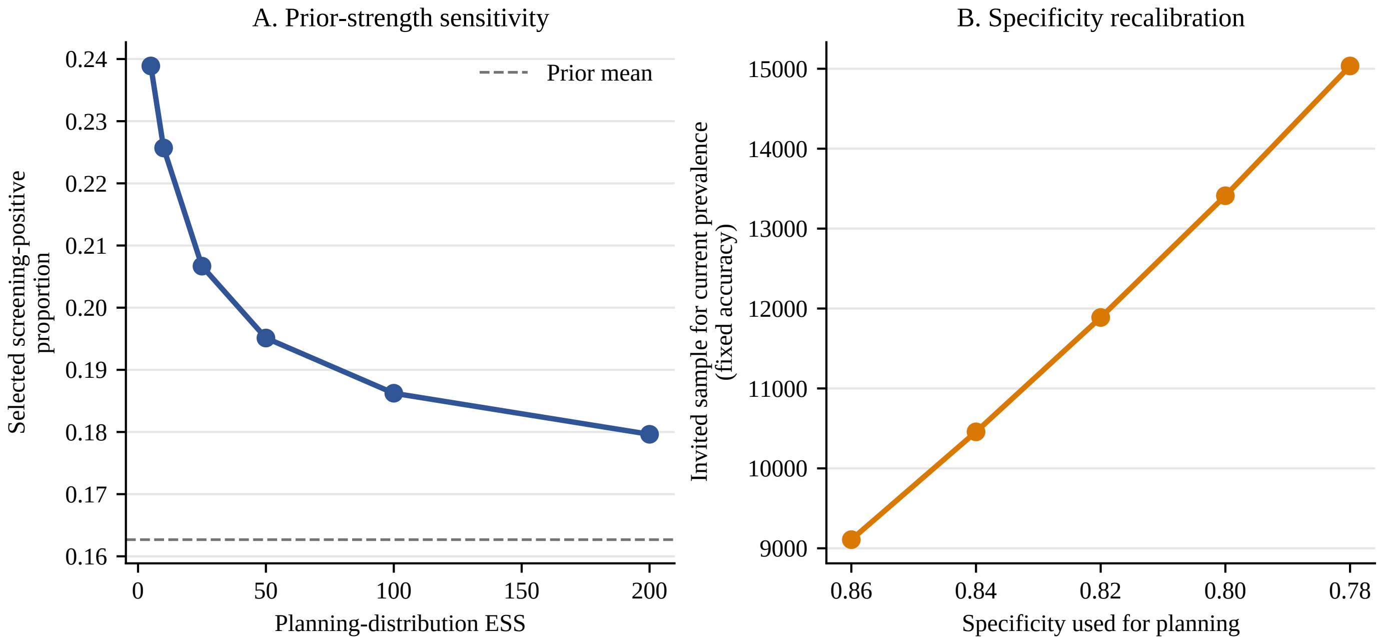

A. Prior-strength sensitivity
Prior mean
Selected screening-positive proportion
0.24
0.23
0.22
0.21
0.20
0.19
0.18
0.17
0.16
0
50
100
150
200
Planning-distribution ESS
B. Specificity recalibration
Invited sample for current prevalence (fixed accuracy)
15000
14000
13000
12000
11000
10000
9000
0.86
0.84
0.82
0.80
0.78
Specificity used for planning
