# Supplementary material for: Decision-theoretic recruitment and validation planning for depression surveys: An illustrative Chinese application
Source: PLoS One. 2026 Oct 1;21(10):e0359867. doi: 10.1371/journal.pone.0359867 (PMC13630222; doi:10.1371/journal.pone.0359867)
Supplement: Supplementary file 4 [file pone.0359867.s002.zip › S2_Reproducibility/results/figure2.pdf]

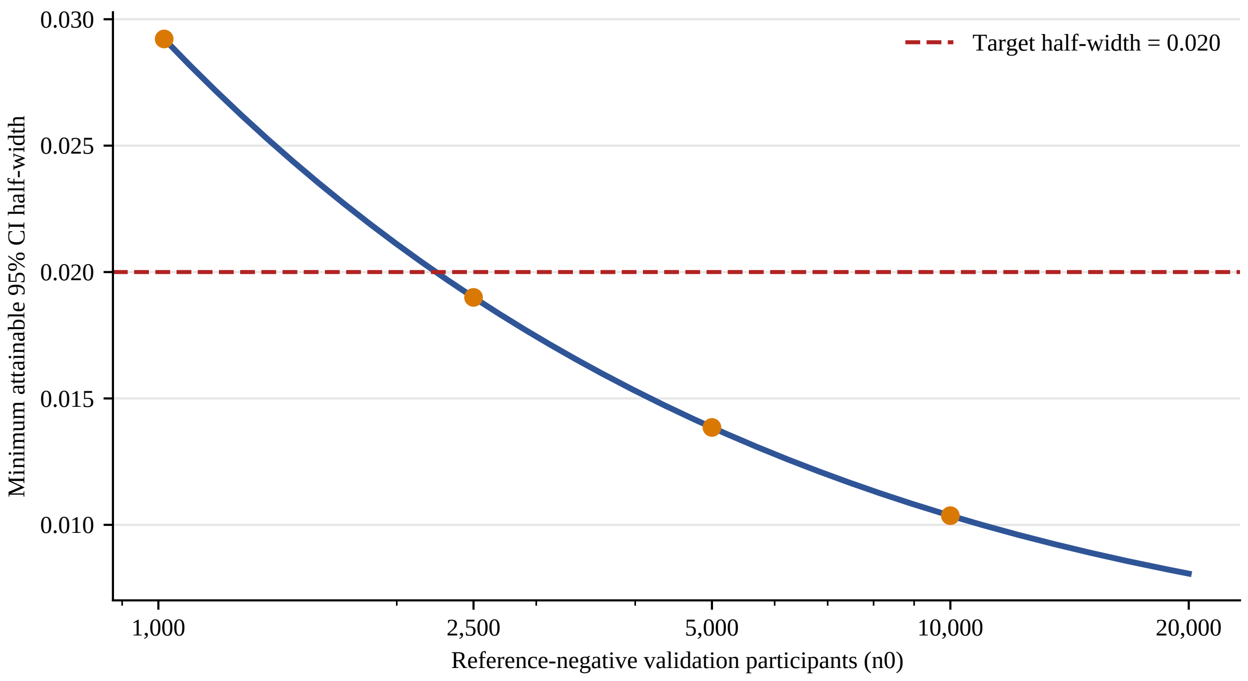
Target half-width = 0.020
Minimum attainable 95% CI half-width
0.030
0.025
0.020
0.015
0.010
1,000
2,500
5,000
10,000
20,000
Reference-negative validation participants (n0)
