# Supplementary material for: Decision-theoretic recruitment and validation planning for depression surveys: An illustrative Chinese application
Source: PLoS One. 2026 Oct 1;21(10):e0359867. doi: 10.1371/journal.pone.0359867 (PMC13630222; doi:10.1371/journal.pone.0359867)
Supplement: Supplementary file 4 [file pone.0359867.s002.zip › S2_Reproducibility/results/figure3.pdf]

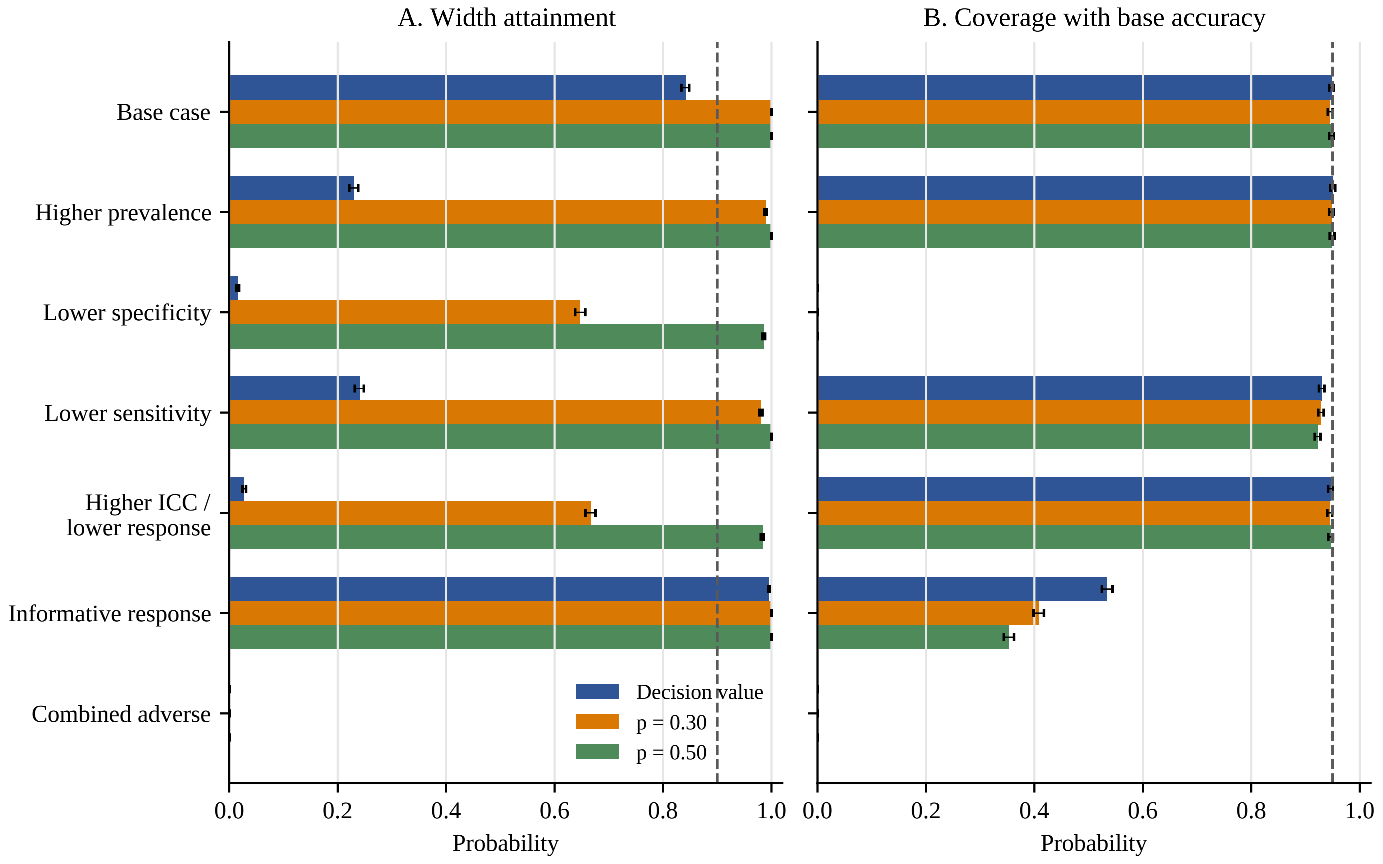
A. Width attainment
B. Coverage with base accuracy
Base case
Higher prevalence
Lower specificity
Lower sensitivity
Higher ICC /
lower response
Informative response
Combined adverse
Decision value
p = 0.30
p = 0.50
0.0
0.2
0.4
0.6
0.8
1.0
Probability
Probability
