# Supplementary material for: Decision-theoretic recruitment and validation planning for depression surveys: An illustrative Chinese application
Source: PLoS One. 2026 Oct 1;21(10):e0359867. doi: 10.1371/journal.pone.0359867 (PMC13630222; doi:10.1371/journal.pone.0359867)
Supplement: Supplementary file 4 [file pone.0359867.s002.zip › S2_Reproducibility/results/figure4.pdf]

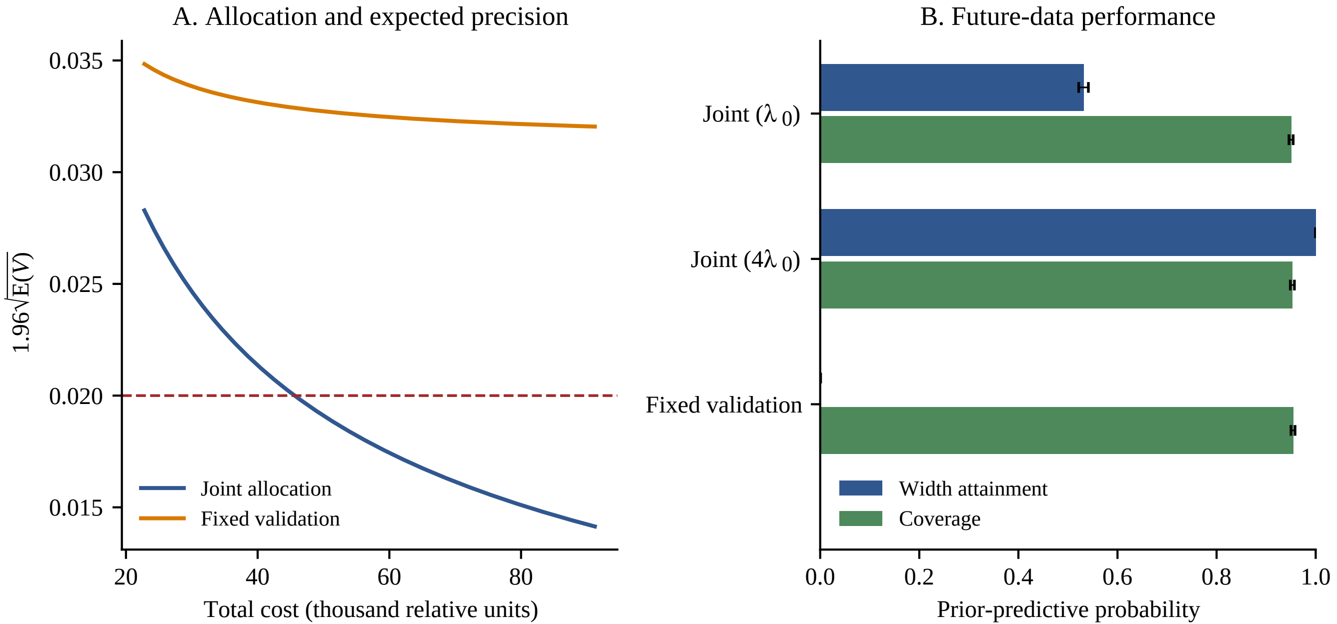

A. Allocation and expected precision
B. Future-data performance
$1.96\sqrt{E(V)}$
0.035
0.030
0.025
0.020
0.015
20
40
60
80
Total cost (thousand relative units)
Joint allocation
Fixed validation
Joint (λ_0)
Joint ($4\lambda_0$)
Fixed validation
Width attainment
Coverage
0.0
0.2
0.4
0.6
0.8
1.0
Prior-predictive probability
