# Supplementary material for: Decision-theoretic recruitment and validation planning for depression surveys: An illustrative Chinese application
Source: PLoS One. 2026 Oct 1;21(10):e0359867. doi: 10.1371/journal.pone.0359867 (PMC13630222; doi:10.1371/journal.pone.0359867)
Supplement: Supplementary file 4 [file pone.0359867.s002.zip › S2_Reproducibility/results/figureS1.pdf]

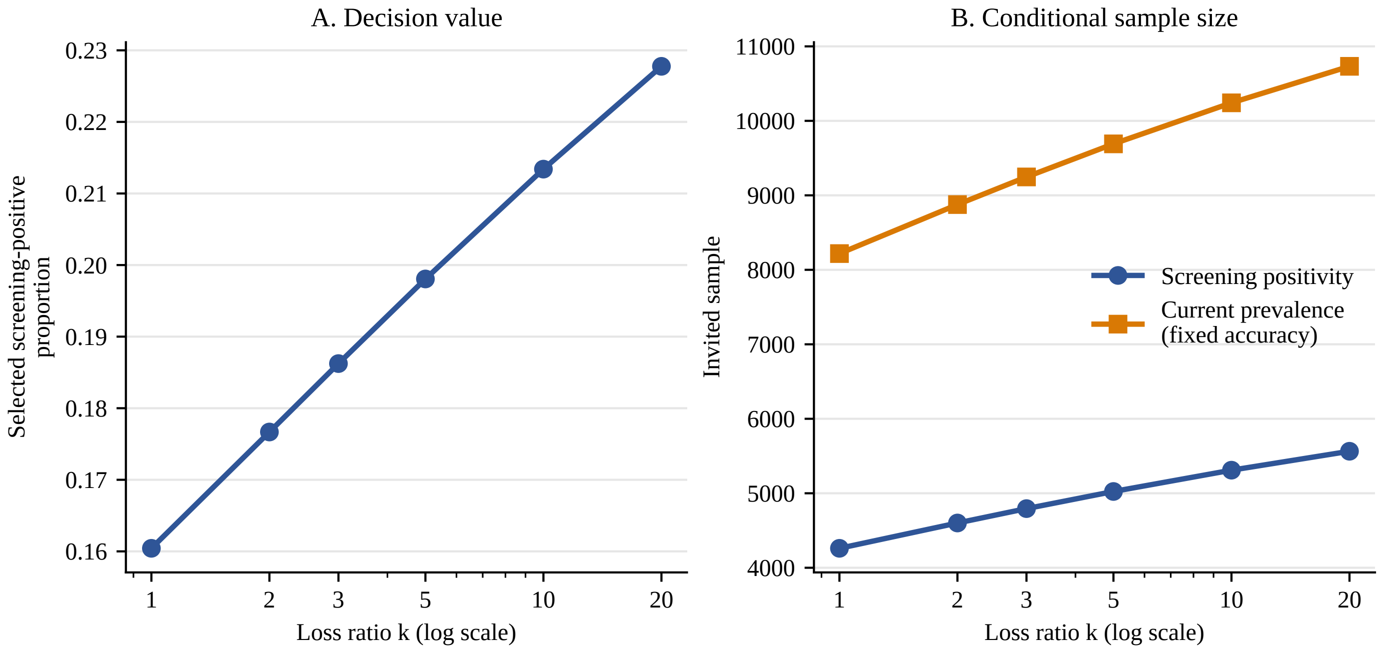
A. Decision value
B. Conditional sample size
Selected screening-positive proportion
0.23
0.22
0.21
0.20
0.19
0.18
0.17
0.16
1
2
3
5
10
20
Loss ratio k (log scale)
Invited sample
11000
10000
9000
8000
7000
6000
5000
4000
Screening positivity
Current prevalence (fixed accuracy)
Loss ratio k (log scale)
