# Supplementary material for: Decision-theoretic recruitment and validation planning for depression surveys: An illustrative Chinese application
Source: PLoS One. 2026 Oct 1;21(10):e0359867. doi: 10.1371/journal.pone.0359867 (PMC13630222; doi:10.1371/journal.pone.0359867)
Supplement: Peer review history

# Peer review: Decision Letter 0

**DOI:** 10.1371/journal.pone.0359867.r001

**Authors:** Jiaci Lin

16 Jul 2026

Dear Dr. liu,

Thank you for submitting your manuscript to PLOS ONE. After careful consideration, we feel that it has merit but does not fully meet PLOS ONE’s publication criteria as it currently stands. Therefore, we invite you to submit a revised version of the manuscript that addresses the points raised during the review process.

Please submit your revised manuscript by Sep 14 2026 11:59PM. If you will need more time than this to complete your revisions, please reply to this message or contact the journal office at plosone@plos.org. When you're ready to submit your revision, log on to https://www.editorialmanager.com/pone/ and select the 'Submissions Needing Revision' folder to locate your manuscript file.

- A letter that responds to each point raised by the academic editor and reviewer(s). You should upload this letter as a separate file labeled 'Response to Reviewers'.
- A marked-up copy of your manuscript that highlights changes made to the original version. You should upload this as a separate file labeled 'Revised Manuscript with Track Changes'.
- An unmarked version of your revised paper without tracked changes. You should upload this as a separate file labeled 'Manuscript'.

If you would like to make changes to your financial disclosure, please include your updated statement in your cover letter. Guidelines for resubmitting your figure files are available below the reviewer comments at the end of this letter.

If applicable, we recommend that you deposit your laboratory protocols in protocols.io to enhance the reproducibility of your results. Protocols.io assigns your protocol its own identifier (DOI) so that it can be cited independently in the future. For instructions see: https://journals.plos.org/plosone/s/submission-guidelines#loc-laboratory-protocols. Additionally, PLOS ONE offers an option for publishing peer-reviewed Lab Protocol articles, which describe protocols hosted on protocols.io. Read more information on sharing protocols at https://plos.org/protocols?utm_medium=editorial-email&utm_source=authorletters&utm_campaign=protocols.

As the corresponding author, your ORCID iD is verified in the submission system and will appear in the published article. PLOS supports the use of ORCID, and we encourage all coauthors to register for an ORCID iD and use it as well. Please encourage your coauthors to verify their ORCID iD within the submission system before final acceptance, as unverified ORCID iDs will not appear in the published article. *Only*  the individual author can complete the verification step; PLOS staff *cannot*  verify ORCID iDs on behalf of authors.

We look forward to receiving your revised manuscript.

Kind regards,

Jiaci Lin

Academic Editor

PLOS One

Journal Requirements:

When submitting your revision, we need you to address these additional requirements.

1. Please ensure that your manuscript meets PLOS ONE's style requirements, including those for file naming. The PLOS ONE style templates can be found at

https://journals.plos.org/plosone/s/file?id=wjVg/PLOSOne_formatting_sample_main_body.pdf and

https://journals.plos.org/plosone/s/file?id=ba62/PLOSOne_formatting_sample_title_authors_affiliations.pdf

2. Please note that PLOS One has specific guidelines on code sharing for submissions in which author-generated code underpins the findings in the manuscript. In these cases, we expect all author-generated code to be made available without restrictions upon publication of the work. Please review our guidelines at https://journals.plos.org/plosone/s/materials-and-software-sharing#loc-sharing-code and ensure that your code is shared in a way that follows best practice and facilitates reproducibility and reuse.

3. Please provide a complete Data Availability Statement in the submission form, ensuring you include all necessary access information. If your research concerns only data provided within your submission, please write "All data are in the manuscript and/or supporting information files" as your Data Availability Statement.

4. Please amend either the abstract on the online submission form (via Edit Submission) or the abstract in the manuscript so that they are identical.

5. We notice that your supplementary tables are included in the manuscript file. Please remove them and upload them with the file type 'Supporting Information'. Please ensure that each Supporting Information file has a legend listed in the manuscript after the references list.

6. Please include captions for your Supporting Information files at the end of your manuscript, and update any in-text citations to match accordingly. Please see our Supporting Information guidelines for more information: http://journals.plos.org/plosone/s/supporting-information.

7. If the reviewer comments include a recommendation to cite specific previously published works, please review and evaluate these publications to determine whether they are relevant and should be cited. There is no requirement to cite these works unless the editor has indicated otherwise.

[Note: HTML markup is below. Please do not edit.]

Reviewers' comments:

Reviewer's Responses to Questions

**Comments to the Author**

1. Is the manuscript technically sound, and do the data support the conclusions?

Reviewer #1: Yes

Reviewer #2: Partly

**********

2. Has the statistical analysis been performed appropriately and rigorously?

Reviewer #1: I Don't Know

Reviewer #2: Yes

**********

3. Have the authors made all data underlying the findings in their manuscript fully available?

The PLOS Data policy

Reviewer #1: Yes

Reviewer #2: Yes

**********

4. Is the manuscript presented in an intelligible fashion and written in standard English?

Reviewer #1: Yes

Reviewer #2: Yes

**********

Reviewer #1: This study presents a potentially valuable methodological framework for sample-size planning in low-prevalence mental health screening surveys. It may have practical implications for public health planning and epidemiological research. However, after careful review, several issues still require further clarification or revision. My specific comments are as follows:

1. The background section should more clearly distinguish between true disorder prevalence and the screening-positive rate.

2. I suggest that the author present the research question earlier and more explicitly. This would help readers grasp the article’s research focus and overall logical flow more quickly.

3. In the discussion, in addition to elaborating on the methodological significance of the proposed methods, the author may also further explore their practical value. For example, the author could describe the contexts in which these methods are applicable and discuss their implications for practice.

4. Some parts of the discussion are repetitive and need to be presented more concisely. In particular, although the discussion of the study’s limitations is comprehensive, it is somewhat list-like. It should be organized more logically.

5. I recommend that the author carefully review and revise the formatting of the entire manuscript, including line spacing and text alignment, in accordance with the journal’s formatting guidelines.

Reviewer #2: - The reported 42.85% sample-size reduction is overstated. Simulations show that p* = 0.173 fails the precision target under higher prevalence, lower specificity or adverse parameters, while p = 0.5 often succeeds; the gain is highly conditional.

- The Beta (15,85) prior relies on only three sources and shifts substantially with moderate heterogeneity, yet guidance for updating is limited.

- The framework depends critically on transportability of Se = Sp = 0.86. When specificity falls to 0.80, precision fails and coverage collapses if original values are assumed; this requires stronger emphasis.

- The cost ratio k = 3 is arbitrary, lacks an elicitation protocol, and materially affects results.

- Equation (8) applies unquantified independence assumptions. The Abstract should qualify results as base-case conditional, the contribution should be framed as integrative, and full code deposition with minor figure/terminology improvements are needed.

**********

what does this mean?). If published, this will include your full peer review and any attached files.

If you choose “no”, your identity will remain anonymous but your review may still be made public.

**Do you want your identity to be public for this peer review?**  For information about this choice, including consent withdrawal, please see our Privacy Policy

Reviewer #1: No

Reviewer #2: No

**********

[NOTE: If reviewer comments were submitted as an attachment file, they will be attached to this email and accessible via the submission site. Please log into your account, locate the manuscript record, and check for the action link "View Attachments". If this link does not appear, there are no attachment files.]

To ensure your figures meet our technical requirements, please review our figure guidelines: https://journals.plos.org/plosone/s/figures

You may also use PLOS’s free figure tool, NAAS, to help you prepare publication quality figures: https://journals.plos.org/plosone/s/figures#loc-tools-for-figure-preparation.

NAAS will assess whether your figures meet our technical requirements by comparing each figure against our figure specifications.

# Author comment: Author response to Decision Letter 1

**DOI:** 10.1371/journal.pone.0359867.r002

24 Jul 2026

Dear Reviewers,

Thank you so much for the opportunity to revise the manuscript. I have addressed the reviewers’ concerns about the estimand, interpretation of the calculated sample-size difference, evidential basis of the Beta prior and loss ratio, transportability of PHQ-9 diagnostic accuracy, and assumptions underlying the sample-size equations. These changes required reanalysis as well as revision of the text.

The primary research question remains unchanged: the manuscript evaluates how prior evidence and asymmetric losses can be used to select a planning screening-positive proportion, p_s^\ast, and quantifies the corresponding invited sample size relative to the conventional p=0.5 calculation in a prespecified Chinese depression-surveillance application. The loss ratio k=c_u/c_o is now treated explicitly as an externally specified policy input that should be elicited before sample-size results are examined. Because the present application contains no such elicitation data, k=3 is retained solely as an illustrative base case and is not described as an empirically optimal value.

The revision addresses each substantive point raised by the editor and reviewers. Reference-standard disorder prevalence π is distinguished throughout from the PHQ-9 screening-positive proportion p_s, with separate sample-size equations for the two estimands. The 42.85% difference is reported only as a fixed-input illustration conditional on k=3 and fixed diagnostic accuracy. Beta(15,85) is treated as an evidence-informed planning distribution with prespecified effective sample size, not as a prior estimated from three independent sources. Uncertainty in sensitivity and specificity is propagated through the delta method, including covariance terms for dependent validation designs. The base value k=3 is identified as illustrative, while six prespecified k values are compared without designating a post hoc “best” value. A prospective loss-elicitation protocol has been added. The assumptions governing clustering, unequal cluster size, non-response, diagnostic transportability, parameter dependence and external or nested validation are also stated and examined explicitly.

All numerical results were recomputed from the accompanying reproducibility scripts. Use of the exact Beta transformation and rounding only after the complete calculation changed the base invited sample from 8,718 to 8,717; the conditional percentage difference remains 42.85% after rounding to two decimal places.

Attachment Submitted filename: Response_to_Reviewers_CORRECTED_v7_2026-07-23.docx

# Peer review: Decision Letter 1

**DOI:** 10.1371/journal.pone.0359867.r003

**Authors:** Jiaci Lin

28 Aug 2026

Dear Dr. liu,

Thank you for submitting your manuscript to PLOS One. After careful consideration, we feel that it has merit but does not fully meet PLOS One’s publication criteria as it currently stands. Therefore, we invite you to submit a revised version of the manuscript that addresses the points raised during the review process.

Please submit your revised manuscript by Sep 26 2026 11:59PM. If you will need more time than this to complete your revisions, please reply to this message or contact the journal office at plosone@plos.org. When you're ready to submit your revision, log on to https://www.editorialmanager.com/pone/ and select the 'Submissions Needing Revision' folder to locate your manuscript file.

- A letter that responds to each point raised by the academic editor and reviewer(s). You should upload this letter as a separate file labeled 'Response to Reviewers'.
- A marked-up copy of your manuscript that highlights changes made to the original version. You should upload this as a separate file labeled 'Revised Manuscript with Track Changes'.
- An unmarked version of your revised paper without tracked changes. You should upload this as a separate file labeled 'Manuscript'.

If you would like to make changes to your financial disclosure, please include your updated statement in your cover letter. Guidelines for resubmitting your figure files are available below the reviewer comments at the end of this letter.

If applicable, we recommend that you deposit your laboratory protocols in protocols.io to enhance the reproducibility of your results. Protocols.io assigns your protocol its own identifier (DOI) so that it can be cited independently in the future. For instructions see: https://journals.plos.org/plosone/s/submission-guidelines#loc-laboratory-protocols. Additionally, PLOS One offers an option for publishing peer-reviewed Lab Protocol articles, which describe protocols hosted on protocols.io. Read more information on sharing protocols at https://plos.org/protocols?utm_medium=editorial-email&utm_source=authorletters&utm_campaign=protocols.

As the corresponding author, your ORCID iD is verified in the submission system and will appear in the published article. PLOS supports the use of ORCID, and we encourage all coauthors to register for an ORCID iD and use it as well. Please encourage your coauthors to verify their ORCID iD within the submission system before final acceptance, as unverified ORCID iDs will not appear in the published article. *Only*  the individual author can complete the verification step; PLOS staff *cannot*  verify ORCID iDs on behalf of authors.

We look forward to receiving your revised manuscript.

Kind regards,

Jiaci Lin

Academic Editor

PLOS One

Journal Requirements:

If the reviewer comments include a recommendation to cite specific previously published works, please review and evaluate these publications to determine whether they are relevant and should be cited. There is no requirement to cite these works unless the editor has indicated otherwise.

Please review your reference list to ensure that it is complete and correct. If you have cited papers that have been retracted, please include the rationale for doing so in the manuscript text, or remove these references and replace them with relevant current references. Any changes to the reference list should be mentioned in the rebuttal letter that accompanies your revised manuscript. If you need to cite a retracted article, indicate the article’s retracted status in the References list and also include a citation and full reference for the retraction notice.

[Note: HTML markup is below. Please do not edit.]

Reviewers' comments:

Reviewer's Responses to Questions

**Comments to the Author**

Reviewer #1: All comments have been addressed

Reviewer #2: All comments have been addressed

**********

2. Is the manuscript technically sound, and do the data support the conclusions?

Reviewer #1: Yes

Reviewer #2: Yes

**********

3. Has the statistical analysis been performed appropriately and rigorously?

Reviewer #1: Yes

Reviewer #2: (No Response)

**********

4. Have the authors made all data underlying the findings in their manuscript fully available?

The PLOS Data policy

Reviewer #1: Yes

Reviewer #2: Yes

**********

5. Is the manuscript presented in an intelligible fashion and written in standard English?

Reviewer #1: Yes

Reviewer #2: Yes

**********

Reviewer #1: The author has responded carefully to the comments raised in the previous round of review, and the revised manuscript has improved substantially.

However, one issue still requires clarification. The manuscript uses the 12-month MDD prevalence estimate, together with the sensitivity and specificity of the PHQ-9, to anchor the planning distribution for the expected PHQ-9 screening-positive proportion. The standard PHQ-9, however, primarily assesses depressive symptoms experienced over the past two weeks. It is therefore unclear whether the PHQ-9 screening-positive proportion and the 12-month reference-standard MDD prevalence are temporally aligned estimands and whether the cited sensitivity and specificity estimates support this mapping.

Reviewer #2: Dear Authors,

The revised version is acceptable and the necessary corrections have been made.

Good luck.

**********

what does this mean?). If published, this will include your full peer review and any attached files.

If you choose “no”, your identity will remain anonymous but your review may still be made public.

**Do you want your identity to be public for this peer review?**  For information about this choice, including consent withdrawal, please see our Privacy Policy

Reviewer #1: No

Reviewer #2: No

**********

[NOTE: If reviewer comments were submitted as an attachment file, they will be attached to this email and accessible via the submission site. Please log into your account, locate the manuscript record, and check for the action link "View Attachments". If this link does not appear, there are no attachment files.]

To ensure your figures meet our technical requirements, please review our figure guidelines: https://journals.plos.org/plosone/s/figures

You may also use PLOS’s free figure tool, NAAS, to help you prepare publication quality figures: https://journals.plos.org/plosone/s/figures#loc-tools-for-figure-preparation.

NAAS will assess whether your figures meet our technical requirements by comparing each figure against our figure specifications.

# Author comment: Author response to Decision Letter 2

**DOI:** 10.1371/journal.pone.0359867.r004

8 Sep 2026

Dear Dr Lin,

Thank you for the further review. The revision replaces the annual-prevalence-based screening anchor and updates the dependent calculations. The principal changes and the response to the remaining temporal-alignment concern are detailed below.

Page and line references identify the PLOS submission edition (Manuscript.docx; 28-page clean rendering) dated 8 September 2026. Supplementary page references identify S1 Appendix. The marked manuscript records changes relative to the uploaded R1 clean manuscript.

Summary of the main revisions

Evidence and outcomes. The screening prior now uses observed PHQ-9 positivity. The 12-month national estimate is retained solely as background, and current MINI-defined prevalence is analysed as a separate outcome. See Methods, pp. 4–6, and S1 Appendix, Section S9, pp. 7–8.

Recalculation and diagnostic validation. Revised inputs were propagated through the sample-size calculations, sensitivity analyses and simulations. Joint recruitment–validation allocation and its operating characteristics are reported separately from conditional planning. See Results, pp. 14–21, Tables 1–5 and Figures 1–4.

Reporting and interpretation. Methods specifies the executed analysis steps; Results follows the calculation sequence; Discussion interprets those results; and the Conclusion answers the study aim. See Methods, pp. 4–14; Discussion, pp. 21–23; and Conclusion, p. 24.

References and reproducibility. References and source-to-input links were checked. The package includes the original-R1 Word comparison, a clean manuscript, the supporting derivations, and executable code with numerical and simulation outputs.

Reviewer 1: the 12-month prevalence and two-week PHQ-9 concern

Comment.

The author has responded carefully to the comments raised in the previous round of review, and the revised manuscript has improved substantially. However, one issue still requires clarification. The manuscript uses the 12-month MDD prevalence estimate, together with the sensitivity and specificity of the PHQ-9, to anchor the planning distribution for the expected PHQ-9 screening-positive proportion. The standard PHQ-9, however, primarily assesses depressive symptoms experienced over the past two weeks. It is therefore unclear whether the PHQ-9 screening-positive proportion and the 12-month reference-standard MDD prevalence are temporally aligned estimands and whether the cited sensitivity and specificity estimates support this mapping.

Response. The original mapping lacked accuracy estimates validated against a 12-month reference target. The correction therefore changes the empirical anchor, outcome definitions and dependent analyses. It is implemented in Methods and Results, with the remaining source limitations identified separately.

1. The annual prevalence estimate was removed from the analysis inputs

The national 12-month MDD estimate no longer enters prior construction, specificity recalibration, parameter draws or simulation scenarios. The Introduction retains the weighted estimate of 2.1% as epidemiological background and corrects its 95% confidence interval to 1.8%–2.4%, as reported in Table 2 of Huang et al. (2019). The prior construction no longer triangulates screening yield from annual prevalence sources.

Location. Introduction, p. 3, lines 37–43; Methods, “Planning prior and updating,” pp. 5–6, lines 107–110; S1 Appendix, Section S9 and Table S7, pp. 7–8.

2. The screening prior was rebuilt from directly observed PHQ-9 results

At PHQ-9 ≥7, the Shanghai validation study reported 170 screening-positive results among 1,045 community participants (Wang et al., 2014, Section 3.3 and Table 1). The revised screening prior uses their observed proportion, 170/1,045 = 0.162679, without converting a disorder-prevalence estimate into screening positivity. Retaining the specified ESS of 100 gives Beta(16.2679426, 83.7320574), replacing Beta(15, 85). ESS remains an investigator-specified discount rather than an estimated national sample size.

Location. Methods, “Planning prior and updating,” pp. 5–6, lines 107–110; Figure 1A; S1 Appendix, Table S7, pp. 7–8; input_provenance.csv.

3. The reference-prevalence analysis was defined separately and indexed to its target

Methods now distinguishes S2w, PHQ-9 positivity for symptoms over the preceding two weeks, from Dcur, the current MINI depression reference classification. Equation (1) uses Se = P(S2w = 1 | Dcur = 1) and Sp = P(S2w = 0 | Dcur = 0). The same study reported 28 MINI-positive participants and threshold-specific sensitivity and specificity of 0.86 (Wang et al., 2014, Sections 3.1 and 3.3; Table 4). These aggregates define the current-reference illustration; current-prevalence parameter draws generate screening positivity from Equation (1).

The source reports PHQ-9 administration followed by MINI assessment and describes the MINI reference in relation to current major depressive episodes (Wang et al., 2014, Sections 2.2–2.3.2). Its two-week follow-up in 100 participants concerned PHQ-9 test–retest reliability (Wang et al., 2014, Section 2.2). That follow-up does not establish the elapsed interval between PHQ-9 and MINI administration, which the source does not specify.

A 12-month prevalence analysis would instead require sensitivity P(S2w = 1 | D12m = 1) and specificity P(S2w = 0 | D12m = 0), where D12m identifies a reference-standard episode during that year. Such annual-reference accuracy was not established by the cited study. The revised numerical analysis therefore concerns directly observed screening positivity and a separate current reference outcome; neither is presented as 12-month MDD prevalence.

Location. Methods, “Estimands and measurement relation,” p. 4, lines 73–84; Equation (1); Methods, “Joint parameter assurance,” p. 10, lines 198–214; S1 Appendix, Section S9, pp. 7–8; Limitations, p. 23, lines 479–486.

4. All dependent numerical results were updated

At the unchanged illustrative loss ratio k = 3, the revised planning proportion is 0.1862346, compared with 0.1727 in R1. Conditional screening invitations increased from 4,519 to 4,794, and current-reference invitations with fixed diagnostic accuracy increased from 8,717 to 9,247. The updated differences from matched p = 0.5 calculations are 39.38%. Integrating uncertainty over the full invitation requirement separately selects 5,346 screening and 11,866 current-reference invitations, with diagnostic accuracy fixed within each current-reference scenario. Sensitivity analyses, simulations, figures, the Abstract and the Conclusion use the revised inputs and outputs.

Location. Results, “Planning value and base-case sample sizes,” p. 14, lines 300–309; Table 1; Results, pp. 15–21; Abstract, p. 2; Conclusion, p. 24, lines 505–513; S1 Appendix, Table S1.

5. The empirical evidence and unresolved source details were made traceable

Table S7 maps each count, diagnostic-accuracy estimate and time-window definition to its source section and analytical use. The directly observed screening mean, 0.162679, is distinguished from the model-implied reference-point probability, 0.159292, calculated using 28/1,045 and rounded Se = Sp = 0.86. Source counts were not altered to force agreement. The source audit also records inconsistencies among published diagnostic summaries. No author-confirmed complete diagnostic table or participant-level dataset is claimed. Sampling uncertainty and population transportability are evaluated separately from numerical rounding.

Location. S1 Appendix, Section S7, p. 5, and Section S9/Table S7, pp. 7–8; Limitations, p. 23, lines 479–486; input_provenance.csv.

Reviewer 2

Comment. “The revised version is acceptable and the necessary corrections have been made.”

Response. Thank you for this assessment. The additional changes prompted by Reviewer 1 have been checked for consistency across the manuscript, supporting analyses and figures.

Further revisions and journal requirements

Methods, decision criteria and validation precision

The study aim is stated at the end of the Introduction. Methods specifies the input distributions, loss ratios, sensitivity grids, random streams, estimators and performance metrics. Conditional recruitment, parameter assurance and simulated interval-width attainment are distinguished. Equations (10)–(11) define the independent-validation variance budget and its feasibility boundary; the reference-point half-width floor is 0.0292, above the 0.020 target. Equations (13)–(15) add decisions over the full recruitment requirement and joint survey–validation allocation, with proofs for the stated objectives in S1 Appendix.

Joint allocation achieved 53.15% prior-predictive width attainment, compared with 0.03% when a nearly equal assumed budget retained the original validation counts. Costs, priors and precision valuations are identified as assumptions. The Discussion distinguishes optimality for the specified risk from attaining a prespecified probability of adequate precision. Operational steps are reported in Methods; response bias, simulation scope and transportability restrictions appear in Limitations.

Location. Introduction, p. 4, lines 66–70; Methods, pp. 7–14; Methods, “Bayes decisions under joint uncertainty” and “Joint allocation of survey and validation samples,” pp. 10–12, lines 215–251; Equations (13)–(15); Table 5 and Figure 4; Discussion, pp. 21–23, lines 436–477; Limitations, pp. 23–24, lines 479–503.

Results, figures, Discussion and Conclusion

Reporting request. “The results section is difficult to follow in its current form. Key findings should be presented more clearly and systematically, with improved figure quality and more transparent reporting.”

Results now proceeds from the planning value and conditional invitation counts to joint parameter assurance, prior sensitivity, diagnostic-validation precision, simulation performance and joint allocation. Tables identify the estimand and precision metric, and the long simulation passages are divided by function. Discussion interprets the corresponding findings. The Conclusion answers the research aim using the recalculated recruitment, validation-allocation and calibration results. Figures 1–4 and S1 are supplied as 600 dpi TIFFs with vector PDF copies; simulation figures include Monte Carlo intervals. Table labels are non-italic, and the full titles of Figures 1 and 2 are bold.

Location. Results, pp. 14–21, Tables 1–5 and Figures 1–4; Discussion, pp. 21–23, lines 436–477; Conclusion, p. 24, lines 505–513.

Reference corrections

All 21 manuscript references were matched to the in-text citations in both directions. Grouped literature citations were replaced by one source per cited sentence. The revised bibliography adds the original PHQ-9 instrument paper, a two-phase prevalence-estimation paper and a study of selective cutoff reporting. Two prevalence reviews used in the former annual-prevalence triangulation and one unused general decision-theory source were removed. Source scope and bibliographic details were checked, including the national prevalence confidence interval and the Shanghai assessment protocol. The PLOS manuscript uses numbered references in order of first appearance; the APA edition uses author–year citations.

Location. Introduction and References; citation_statement_map.csv; citation_audit.csv; S1 Appendix, Table S7.

Submission files and reproducibility

The package supplies separate files labelled Response to Reviewers, Revised Manuscript with Track Changes and Manuscript. The native Word comparison uses the original clean R1 manuscript corresponding to pages 8–33 of the uploaded submission PDF. Accepting its content revisions reproduces the current clean manuscript; rejecting them restores the R1 content. The comparison is reconstructed from the two versions and retains the revised page layout.

S1 Appendix supplies the derivations, simulation specifications and source mapping. S2_Reproducibility.zip contains the executed analysis script, software requirements, numerical outputs and replicate arrays. The computational record reproduces the declared aggregate-input illustration and distinguishes simulated observations from participant data. The funding and competing-interest declarations are retained.

Location. Data and code availability, p. 25, lines 521–526; Declarations; S1 Appendix; S2_Reproducibility; original_comparison.json.

Sincerely,

Han Liu

Attachment Submitted filename: Response_to_Reviewers(8).docx

# Peer review: Decision Letter 2

**DOI:** 10.1371/journal.pone.0359867.r005

**Authors:** Jiaci Lin

20 Sep 2026

Decision-theoretic recruitment and validation planning for depression surveys: an illustrative Chinese application

PONE-D-26-26922R2

Dear Dr. liu,

We’re pleased to inform you that your manuscript has been judged scientifically suitable for publication and will be formally accepted for publication once it meets all outstanding technical requirements.

Within one week, you’ll receive an e-mail detailing the required amendments. When these have been addressed, you’ll receive a formal acceptance letter and your manuscript will be scheduled for publication.

An invoice will be generated when your article is formally accepted. Please note, if your institution has a publishing partnership with PLOS and your article meets the relevant criteria, all or part of your publication costs will be covered. Please make sure your user information is up-to-date by logging into Editorial Manager at Editorial Manager® and clicking the ‘Update My Information' link at the top of the page. For questions related to billing, please contact billing support.

If your institution or institutions have a press office, please notify them about your upcoming paper to help maximize its impact. If they’ll be preparing press materials, please inform our press team as soon as possible -- no later than 48 hours after receiving the formal acceptance. Your manuscript will remain under strict press embargo until 2 pm Eastern Time on the date of publication. For more information, please contact onepress@plos.org.

Kind regards,

Jiaci Lin

Academic Editor

PLOS One

Additional Editor Comments (optional):

Reviewers' comments:

Reviewer's Responses to Questions

**Comments to the Author**

Reviewer #1: All comments have been addressed

**********

2. Is the manuscript technically sound, and do the data support the conclusions?

Reviewer #1: Yes

**********

3. Has the statistical analysis been performed appropriately and rigorously?

Reviewer #1: I Don't Know

**********

4. Have the authors made all data underlying the findings in their manuscript fully available?

The PLOS Data policy

Reviewer #1: Yes

**********

5. Is the manuscript presented in an intelligible fashion and written in standard English?

Reviewer #1: Yes

**********

Reviewer #1: The revised manuscript is acceptable. The author have carefully addressed all the comments and suggestions raised during the previous review stage.

**********

what does this mean?). If published, this will include your full peer review and any attached files.

If you choose “no”, your identity will remain anonymous but your review may still be made public.

**Do you want your identity to be public for this peer review?**  For information about this choice, including consent withdrawal, please see our Privacy Policy

Reviewer #1: No

**********

# Editor report: Acceptance letter

**DOI:** 10.1371/journal.pone.0359867.r006

**Authors:** Jiaci Lin

PONE-D-26-26922R2

PLOS One

Dear Dr. liu,

I'm pleased to inform you that your manuscript has been deemed suitable for publication in PLOS One. Congratulations! Your manuscript is now being handed over to our production team.

At this stage, our production department will prepare your paper for publication. This includes ensuring the following:

* All references, tables, and figures are properly cited

* All relevant supporting information is included in the manuscript submission,

* There are no issues that prevent the paper from being properly typeset

You will receive further instructions from the production team, including instructions on how to review your proof when it is ready. Please keep in mind that we are working through a large volume of accepted articles, so please give us a few days to review your paper and let you know the next and final steps.

Lastly, if your institution or institutions have a press office, please let them know about your upcoming paper now to help maximize its impact. If they'll be preparing press materials, please inform our press team within the next 48 hours. Your manuscript will remain under strict press embargo until 2 pm Eastern Time on the date of publication. For more information, please contact onepress@plos.org.

You will receive an invoice from PLOS for your publication fee after your manuscript has reached the completed accept phase. If you receive an email requesting payment before acceptance or for any other service, this may be a phishing scheme. Learn how to identify phishing emails and protect your accounts at https://explore.plos.org/phishing.

If we can help with anything else, please email us at customercare@plos.org.

Thank you for submitting your work to PLOS One and supporting open access.

Kind regards,

PLOS One Editorial Office Staff

on behalf of

Dr. Jiaci Lin

Academic Editor

PLOS One
